# Spatial transcriptome and single-cell sequencing reveal the role of nucleotide metabolism in breast cancer progression and tumor microenvironment

**DOI:** 10.3389/fonc.2025.1703778

**Published:** 2026-01-14

**Authors:** Yuejun Pan, Yong Xu, Ke Gong, Songlin Yuan

**Affiliations:** Department of Breast and Thyroid Surgery, Changde Hospital, Xiangya School of Medicine, Central South University (the first People’s Hospital of Changde City), Changde, Hunan, China

**Keywords:** breast cancer, immunotherapy, machine learning, nucleotide metabolism, single-cell sequencing

## Abstract

**Background:**

The complexities of nucleotide metabolism in breast cancer (BC) cells are not yet fully understood. A deeper exploration of the various tumor subpopulations and their interactions with the tumor microenvironment (TME) could provide important insights into these clinically relevant signaling pathways.

**Methods:**

We integrated five distinct single-cell enrichment scoring methodologies to conduct a comprehensive enrichment analysis of BC cell populations. The analytical findings underwent subsequent validation using an independent single-cell cohort. Tumor cell clusters were categorized based on their average enrichment scores. Functional analyses were carried out using several tools, including CellChat, Monocle, CopyKAT, SCENIC, and CytoTRACE. The RCTD method was then employed to map the single-cell clusters onto spatial transcriptomics data, facilitating the evaluation of cellular dependencies and pathway activities to differentiate tumor cell subtypes. A prognostic framework was subsequently established using large-scale transcriptomic datasets, enabling prediction of immunotherapy responsiveness. Experimental validation further confirmed expression patterns of pivotal genes implicated in therapeutic outcomes.

**Results:**

Tumor cells exhibit significantly upregulated nucleotide metabolic activity, enabling their classification into two distinct subgroups: NUhighepi and NUlowepi. Cells within the NUhighepi subgroup demonstrate pronounced malignant phenotypes. Intercellular communication analysis performed with the stLearn platform revealed robust interactions between NUhighepi cells and fibroblasts. Supporting this finding, spatial transcriptomic analysis via MISTy revealed a distinct dependency of NUhighepi on fibroblasts. A robust prognostic model, developed using various machine learning algorithms, was able to predict survival outcomes and responses to immunotherapy. Furthermore, targeted drugs were identified for both the high and low scoring groups. Experimental investigations confirmed the expression of core genes in different breast cancer cells.

**Discussion:**

In conclusion, we developed a nucleotide metabolism-derived prognostic signature for BC, with DCTPP1 highlighted as a promising biomarker and therapeutic target. These findings provide a valuable framework for early clinical intervention and show promising potential for predicting responses to immunotherapy in BC patients.

## 1 Introduction

Breast cancer (BC) continues to be the leading malignancy among women globally, characterized by elevated incidence and mortality rates. A recent report from the American Cancer Society indicates that BC represents 31% of all new cancer diagnoses in women (1). Although treatment advancements, including surgery, chemotherapy, targeted therapy, endocrine therapy, and immunotherapy, have significantly improved the overall 5-year survival rate to 90% for patients diagnosed with localized early-stage BC, the survival rate for those with advanced BC remains under 30% (2). Additionally, drug resistance continues to be a major obstacle to the effectiveness of therapies in certain BC cases. Since BC progression is driven by a complex multi-gene process, more precise strategies are essential to address the limitations of conventional therapies. The integration of genomic, transcriptomic, and proteomic research provides a deeper understanding of the unique biological features of tumors, offering valuable insights for evaluation and identifying novel diagnostic methods and therapeutic targets for BC patients.

Tumor progression is often linked to dysregulated metabolic profiles, wherein metabolic reprogramming serves a pivotal function in driving oncogenesis. A central element within these alterations involves nucleotide metabolism (3). This process encompasses multiple enzymes that regulate the synthesis and degradation of nucleotides, covering both purine and pyrimidine metabolic pathways that are indispensable for the production of DNA and RNA. Nucleotide biosynthesis proceeds via *de novo* routes as well as salvage mechanisms that recycle nucleosides and nucleobases (4, 5). Emerging evidence indicates that hyperactive nucleotide metabolic pathways contribute significantly to tumor growth, resistance to chemotherapeutic agents, metastatic behavior, and immune escape (6). Consequently, therapeutic approaches exploiting the metabolic vulnerabilities of tumor cells have gained significant attention as promising strategies. A representative case is 5-fluorouracil, which functions by targeting thymidylate synthase. This enzyme is essential for catalyzing the *de novo* biosynthesis of 2′-deoxythymidine-5′-monophosphate (dTMP), and its inhibition has proven highly valuable in clinical oncology (7). In addition to serving as essential substrates for nucleic acid synthesis, nucleotides also support various processes that are hyperactive in cancer cells. As a result, targeting nucleotide metabolism offers significant potential as part of combination therapies, an approach that remains largely underexplored.

In numerous cancers, the metabolic pathways responsible for nucleotide production are frequently enhanced to support the rapid and uncontrolled proliferation of malignant cells (8). This elevated metabolic activity, however, may also induce genetic instability, thus further promoting oncogenesis and disease progression (9). A central regulator of nucleotide biosynthetic processes is the proto-oncogene *MYC*, which critically modulates the transcription of several rate-limiting enzymes. These include the multifunctional CAD protein, thymidylate synthase (TS), and inosine monophosphate dehydrogenase (IMPDH), all of which contribute significantly to metabolic reprogramming in tumors (10). Notably, targeting nucleotide metabolism presents a direct strategy to reverse tumor-induced immunosuppression. This is particularly relevant given that extracellular purines released from malignant cells can engage with inhibitory receptors present on immune cells (11). Furthermore, studies indicate that combining agents that disrupt nucleotide metabolism with immunotherapeutic regimens can produce synergistic anti-tumor outcomes (12). Thus, modulating nucleotide metabolic pathways is emerging as a viable therapeutic approach to either directly eradicate malignant cells or potentiate existing oncological treatments.

Nucleoside analog drugs, including Gemcitabine and Capecitabine, constitute a critical component in the clinical management of BC and various other cancers. Evidence suggests that elevated pyrimidine nucleotide levels after chemotherapy may represent a promising metabolic target for improving therapeutic responses in triple-negative BC (TNBC). Accordingly, pharmacological suppression of *de novo* pyrimidine synthesis enhances the susceptibility of TNBC cells to genotoxic chemotherapeutic agents, leading to augmented DNA damage (13). Nevertheless, robust and clinically applicable predictive models centered on nucleotide metabolism remain underdeveloped for BC, limiting accurate prognosis and treatment evaluation. Enhancing both overall and disease-free survival continues to be a central objective in oncology. Hence, the construction of individualized prognostic frameworks based on nucleotide metabolism-associated genes (NMRGs) derived from BC clinical specimens is imperative for refining predictive accuracy and guiding treatment strategies.

Although the crosstalk within the tumor-immune microenvironment has been well-documented, the intrinsic diversity of cancer cells poses significant challenges to the precise delineation of these populations, underscoring the need for more in-depth analyses. In this study, we utilized multiomics approaches, including scRNA-seq and Spatial transcriptomics (ST), to comprehensively explore the communication between tumor and immune cells in breast cancer. We focused specifically on tumor cells with elevated nucleotide metabolism (NUhighepi), a previously underexplored aspect identified within BC tissues. Our findings indicate that the tumor microenvironment (TME) significantly reshapes the biological properties of NUhighepi cells, enhancing their capacity to promote tumor progression. We are the first to report a robust correlation between NUhighepi populations and fibroblast associated with tumors. Specifically, this crosstalk activates pro-oncogenic signaling within fibroblasts, thereby amplifying the invasive behavior of NUhighepi cells and accelerating malignant progression. These observations contribute novel mechanistic understanding regarding nucleotide metabolism in oncogenesis.

## 2 Methods and materials

### 2.1 Transcriptome data acquired and processing

Transcriptomic profiling data, gene mutations, and comprehensive clinical metadata for breast cancer were retrieved from the TCGA database (n=1095) and used for model construction. Additionally, the GEO expression profiles from GSE20685 (n = 327) and GSE88770 (n = 117) were downloaded to serve as an external independent validation cohort to assess the model’s stability and accuracy. Data preprocessing followed a standardized protocol to ensure cross-platform compatibility and minimize technical variation. All transcriptomic data were converted to TPM values and subjected to log2 transformation to ensure suitability for downstream analyses. Batch effect adjustments between TCGA-BRCA, GSE20685, and GSE88770 were performed using the “sva” package (14). All cohorts used in this article were described in **Supplementary Table S1**.

### 2.2 Clarification of tumor metabolic heterogeneity

To elucidate tumor metabolic heterogeneity, we performed metabolic pathway activity profiling via single-sample Gene Set En Enrichment Analysis (ssGSEA) implemented in the R package “GSVA”. The differential expressions of 162 metabolism pathways which downloaded from the MSigDB database (https://www.gsea-msigdb.org/gsea/msigdb) between normal and tumor tissues were calculated using the “Limma” package.

### 2.3 Single-cell RNA sequencing analysis

Single-cell data for BC were retrieved from the GEO database, specifically from the GSE161529 dataset, which includes ten samples. Quality control of the scRNA-seq data was performed using the “seurat” package. To ensure high-quality scRNA-seq data, filtering criteria included the retention of cells exhibiting mitochondrial gene content below 10%, more than 200 detected genes per cell, and features detected in a minimum of three cells with expression levels ranging from 200 to 7000 counts. This process resulted in the selection of 50, 917 qualified cells for subsequent analyses. The retained cells then underwent scaling and normalization via a linear regression approach employing log-normalization. Following normalization, the 2, 000 most variable genes were identified using the “FindVariableFeatures” method. Finally, integration and scaling of the dataset were performed with the “IntegrateData” and “ScaleData” functions, respectively.

Dimensionality reduction was initially performed by applying principal component analysis (PCA) to establish anchor points. Subsequently, the t-distributed stochastic neighbor embedding (t-SNE) algorithm was applied to visualize the first 20 principal components (PCs), facilitating the detection of biologically relevant clusters. Inter-cluster variations in cell cycle phase distribution were further examined using established cell cycle markers integrated within the “Seurat” toolkit.

In addition, we downloaded single-cell cohorts of five additional cancer types from the TISCH2 portal (http://tisch.comp-genomics.org/) for pan-cancer analysis. The standard scRNA-Seq processing workflow—including quality control, normalization, unsupervised clustering, and cell type annotation—had already been completed by the TISCH2 platform.

### 2.4 Cell type recognition

To pinpoint cluster-specific marker genes, we performed genome-wide differential expression profiling across all identified cell clusters. This analysis was carried out using Seurat’s FindAllMarkers function (15), applying thresholds of an adjusted P-value < 0.05, expression proportion > 0.25, and |log_2_FC| > 0.25 (**Supplementary Table 2**). Following this, distinct cell clusters were identified and annotated using the singleR package, based on the composition patterns of the marker genes. These annotations were manually verified and corrected as necessary, with validation provided by the CellMarker database.

### 2.5 Gene set score

An integrated approach incorporating five different scoring algorithms was employed through the irGSEA package (accessible at https://github.com/chuiqin/irGSEA), leveraging single-cell RNA sequencing data. The function irGSEA.score was executed under the following parameter configuration: the min.cells parameter was defined as 3, min.feature was assigned a value of 0, custom was disabled (set as FALSE), msigdb remained enabled (TRUE), the species designated as “Homo sapiens”, and the kernel density estimate function (kcdf) specified as ‘Gaussian’.

### 2.6 Cell-cell communication analysis

To investigate intercellular communication across distinct cell populations within the tumor microenvironment, we applied the “CellChat” package (v1.6.1) (16) and subsequently reconstructed the cell communication network. The netVisual_diffInteraction function was applied to graphically represent variations in interaction strength among cells, whereas the identifyCommunicationPatterns function facilitated the detection of recurrent signaling motifs. Leveraging the CellChatDB reference database, major signaling cascades and ligand–receptor interactions were discerned. A statistical significance threshold (*p* < 0.05) was adopted to determine the robustness of inferred cell–cell communication events.

### 2.7 Constructing single-cell trajectories in BC

Single-cell trajectory reconstruction was conducted with Monocle2 (v2.16.0), an R package designed to model cellular transitions by representing high-dimensional transcriptomic states along a unidimensional pseudotemporal axis (17). Epithelial cell clusters were imported into R, and a CellDataSet object was initialized using the newCellDataSetfunction under the negbinomial.sizeexpression family. Genes selected for trajectory inference satisfied the thresholds of mean_expression ≥ 0.1 and dispersion_empirical ≥ 1 * dispersion_fitas determined by the package. Dimensionality reduction was performed via the reduceDimensionfunction, applying the “DDRTree” algorithm with max_components = 2. The resulting trajectories were visualized using plot_cell_trajectoryto display a minimum spanning tree of cells. Genes displaying significant variation across pseudotime (q-value < 1×10^-5^), identified through differentialGeneTest, were visualized in a heatmap via plot_pseudotime_heatmapand clustered into co-expression modules.

### 2.8 Cancer cell prediction

The CopyKAT package (18) was employed to predict cancer cells. Endothelial cells from the normal group were used as a reference, and epithelial cells was extracted for the prediction process. Cells exhibiting significant aneuploidy mutations were classified as cancer cells. These predicted cancer cells were subsequently annotated as a new cell type for downstream analysis.

### 2.9 CytoTRACE analysis

The computational framework CytoTRACE enables accurate inference of cellular differentiation states from scRNA-seq data. Validation across extensive datasets confirms its superior performance over conventional methods in assessing cellular stemness (19). Using the R implementation (v0.3.3), CytoTRACE scores were computed for malignant populations, with values spanning 0 to 1. Elevated scores correlate with enhanced stemness (reduced differentiation), whereas diminished scores indicate a more differentiated phenotype.

### 2.10 Spatial transcriptome data collection and preprocessing

Breast cancer spatial transcriptomics (ST) data were acquired from the GEO database (https://www.ncbi.nlm.nih.gov/geo/) and 10x Genomics official website (https://www.10xgenomics.com/). Three representative samples were selected, including two 10x platform datasets (BRCA2 and BRCA3) and sample GSM6177603 from the GSE203612 cohort (20). All three samples are 10x Genomics data. Data processing was performed using the Seurat package, where the SCT transformation method was applied for normalization. Key functions including SelectIntegrationFeatures and IntegrateData were used for data integration, followed by unsupervised clustering to identify spatially coherent regions. Cell population annotations were determined by integrating H&E staining patterns with highly variable genes in each cluster, visualized via SpatialDimPlot.

### 2.11 Cell type decomposition analysis of spatial transcriptome data

For accurate cell-type identification in spatial transcriptomic data, we implemented the Robust Cell Type Decomposition (RCTD) computational framework. The methodology established correspondence between single-cell RNA sequencing reference data and spatial profiling information (21), supporting subsequent cell type-specific analyses. Cell type-specific marker genes were identified through the Seurat FindAllMarkers function, prioritizing markers demonstrating positive expression differences as indicated by log2-transformed fold change values. The standard analytical workflow of RCTD was subsequently implemented in its complete doublet mode, integrating both reference scRNA-seq data and Visium spatial transcriptomics information.

### 2.12 Spatial trajectory analysis and cell-cell interaction

Cellular communication networks were initially characterized using the stLearn computational toolkit implemented in Python (22), which systematically integrates spatial proximity data with ligand-receptor co-expression patterns. For trajectory reconstruction, we implemented Partition-based Graph Abstraction (PAGA) to generate a k-nearest neighbor graph (k=15, based on top 50 principal components) from transcriptionally distinct cellular states identified through Leiden clustering (resolution=1.0). Spatial coordinates were incorporated as topological constraints to preserve tissue architecture integrity. Pseudotemporal ordering was subsequently established using the Spatial Morphology Eigenvector (SME) algorithm, which performs spectral decomposition on a spatially-smoothed matrix (Gaussian kernel, bandwidth=100μm) to project cells onto principal variation components. Statistical significance was rigorously validated through permutation testing (n=1000 iterations).

### 2.13 Construction of prognostic signature by integrative machine learning approaches

To construct a robust nucleotide metabolism-related prognostic model with high predictive accuracy, we implemented an integrative machine learning framework encompassing 10 algorithmic categories with 101 specific combinations (23). The selected methodologies included stepwise Cox, random survival forest (RSF), elastic network (Enet), Lasso, Ridge, CoxBoost, supervised principal components (SuperPC), generalized boosted regression modeling (GBM), partial least squares regression for Cox (plsRcox), and survival support vector machine (survival-SVM).

The procedure for generating the signatures involved the following steps: (a) Identification of differentially expressed genes (DEGs) between NUhighepi and NUlowepi cells using FindMarker, followed by univariate Cox regression to select prognosis-associated genes (*p* < 0.05) from TCGA cohort; (b) Construction of prognostic models through 101 machine learning combinations with LOOCV and ten-fold cross-validation; (c) Development of a multivariable Cox model based on nucleotide metabolism-related genes to generate nucleotide metabolism-related score (NMRS); (d) External validation using GSE20685 and GSE88770 cohorts; (e) Model optimization based on Harrell’s C-index. The final NMRS signature was evaluated through time-dependent ROC analysis and Cox regression.

### 2.14 Analysis of genomic variation between NMRS risk subgroups

The Mutant Allele Tumor Heterogeneity (MATH) score serves as a quantitative metric for assessing intratumor heterogeneity (ITH) by evaluating the distribution of mutant allele frequencies. This score is computed using whole-exome sequencing data obtained from tumor tissues and paired normal samples, offering an objective measure of ITH (24). Its prognostic value has been established across multiple cancer types, such as head and neck squamous cell carcinoma, colorectal cancer, and renal cell carcinoma (25). In the present investigation, the MATH scores were calculated for samples from the TCGA-BRCA cohort. The analysis utilized whole-exome sequencing data obtained from the Genomic Data Commons (GDC) Data Portal (https://portal.gdc.cancer.gov/), under the TCGA-BRCA project, which includes tumor and matched normal samples from 1, 085 patients. The calculation was performed using the inferHeterogeneity function within the maftoolsR package, following established methodologies (26). Subsequent survival analyses were then carried out based on the derived MATH scores. Mutational patterns linked to NMRS stratification were analyzed through maftools-generated waterfall plots, systematically comparing somatic alterations between high and low NMRS cohorts. Parallel investigation of copy number alterations focused on the 30 most differentially expressed genes identified through NMRS-based grouping.

### 2.15 Integrated profiling of immune features and response to immune checkpoint inhibition​

A comparative assessment of immune checkpoint expression patterns among these subgroups was performed using the ESTIMATE R package to determine immune and stromal scores within tumor tissues (27). Immunocyte infiltration relationships with NMRS were characterized through CIBERSORT-based quantitation of 22 lymphocyte subpopulations, with technical reproducibility confirmed by ssGSEA. The anti-cancer immunity cycle, which comprises seven essential steps—including cancer antigen release, antigen presentation, immune cell priming and activation, trafficking to tumor sites, infiltration into tumors, T cell-mediated cancer cell recognition, and eventual cancer cell elimination—plays a fundamental role in immuno-oncology. Activity scores for each step were obtained for TCGA-BRCA samples from the Tracking Tumor Immunophenotype (TIP) platform (http://biocc.hrbmu.edu.cn/TIP/index.jsp). The Immunophenoscore (IPS) data for the TCGA-BRCA cohort were obtained from The Cancer Immunome Atlas (TCIA, https://tcia.at/home) to evaluate immunogenicity. These precomputed scores incorporate multiple immunogenic factors including immunomodulators, immunosuppressive cells, MHC molecules, and effector cells, where higher scores indicate better predicted response to immunotherapy. Furthermore, the Tumor Immune Dysfunction and Exclusion (TIDE) computational framework (http://tide.dfci.harvard.edu/) was utilized to derive TIDE, dysfunction, and exclusion scores for samples within the TCGA-BRCA cohort, with subsequent comparison of these metrics between groups (28). The TIDE score functions as a computational proxy for tumor immune microenvironment activity, quantifying the extent of immune evasion. Dysfunction scores, calculated from the expression signatures of T-cell exhaustion-associated genes, indicate the loss of effector function in tumor-infiltrating T cells under persistent antigen exposure. The exclusion score assesses the establishment of an immune-excluded tumor microenvironment by evaluating gene sets implicated in stromal fibrosis, aberrant angiogenesis, and other mechanisms that impede immune cell recruitment. Differences in the proportion of patients exhibiting a response to immunotherapy between cohorts were evaluated using the chi-square test.

### 2.16 Assessment of immunotherapeutic response

Immunotherapy data were obtained from the IMvigor210 cohort (29) and the Tumor Immunotherapy Gene Expression Resource (TIGER) database (25) which includes multiple immunotherapy cohorts with detailed clinical annotations: Melanoma-GSE78220 (30), Melanoma-GSE91061 (31), Melanoma-PRJEB23709 (32), RCC-Braun_2020 (33). The IMvigor210 cohort was accessed using the “IMvigor210CoreBiologies” R package, while all other cohorts were obtained directly from the TIGER portal. These datasets provide comprehensive gene expression profiles, survival information, and immunotherapy response data, enabling robust validation of the NMRS’s predictive capacity for immunotherapy efficacy. Following the same computational pipeline, all cohorts were stratified into High-NMRS and Low-NMRS subgroups. The predictive performance of NMRS was subsequently evaluated through survival analyses correlating NMRS stratification with clinical outcomes across these independent immunotherapy cohorts.

### 2.17 Predicting potential drugs for high-NMRS scores breast cancer patients

To discover potential therapeutics for breast cancer patients with elevated NMRS scores, we systematically integrated pharmacogenomic data from multiple sources. Initial analysis utilized the Genomics of Drug Sensitivity in Cancer (GDSC) database to calculate drug IC50 values in breast cancer samples. Additionally, we specifically examined the correlation between IC50 values of standard-of-care endocrine and targeted therapies and NMRS scores to explore differences in predicted sensitivity between high- and low NMRS subgroups. We further investigated correlations between NMRS scores and the efficacy of standard endocrine and targeted therapies. Expanded screening employed drug response data from CTRP v2.0 and PRISM Repurposing datasets (34). Following removal of duplicates and compounds with significant missing values, 481 CTRP and 1448 PRISM compounds were analyzed. Predictive AUC values for breast cancer patients were generated using ridge regression models trained on CCLE gene expression and drug sensitivity data. To identify high NMRS specific candidate compounds, we stratified patients based on their NMRS scores and conducted differential drug response analysis between the top decile (high NMRS) and bottom decile (low NMRS) groups. Compounds demonstrating enhanced sensitivity in the high NMRS group (statistical significance *p* < 0.05) were prioritized for subsequent analysis. To validate the robustness of these findings, we conducted Spearman’s rank correlation analysis evaluating the association between drug response (AUC values) and NMRS scores across the complete sample set. Following this assessment, agents exhibiting negative correlation coefficients (R < -0.2) were designated as high-confidence candidates for further investigation.

### 2.18 Cell culture, transfection and infection

The human normal mammary epithelial cell line MCF-10A (CL-0525) along with breast cancer lines MCF-7 (CL-0149), SK-BR3 (CL-0211), and MDA-MB-231 (CL-0150B) were acquired from Procell (Wuhan, China). Cells were maintained in Dulbecco’s Modified Eagle Medium (FUSHENBIO, Shanghai) containing 10% fetal bovine serum (FUSHENBIO, Shanghai), under standard culture conditions (37 °C, 5% CO_2_).Tumor cells were seeded into 6-well plates and incubated overnight before transfection with small interfering RNA (siRNA). And Commercial siRNAs were purchased from Sigma-Aldrich (si-DCTPP1-1: SASI_Hs01_00014964, si-DCTPP1-2: SASI_Hs01_00014965).

### 2.19 Quantitative real-time reverse transcription PCR and western blotting

Quantitative real-time PCR (qRT–PCR) was performed to measure mRNA expression levels in cellular samples. Total RNA extraction was carried out with TRIzol^®^ reagent (Takara, Shiga, Japan), followed by cDNA synthesis using the PrimeScript™ RT reagent kit (Takara) in accordance with the provided protocol. Relative gene expression between target and reference samples was quantified via the 2−ΔΔCt algorithm, which identifies the amplification cycle at which fluorescence exceeds the system-defined threshold (35). This approach enabled the assessment of relative transcript abundance for pivotal genes implicated in nucleotide metabolism. Glyceraldehyde-3-phosphate dehydrogenase (GAPDH) was employed as the endogenous control for normalization. All primer sequences used are provided in **Supplementary Table 3**. Protein extraction was conducted with ice-cold lysis buffer containing inhibitors, followed by BCA quantification. Samples were resolved on 4–12% SDS-PAGE gels, transferred to PVDF membranes, blocked, and probed with antibodies. Detection was performed using chemiluminescent substrate. The antibodies used in this study include: DCTPP1 (Proteintech, # 16684-1-AP, 1:1000), β-Actin (Proteintech, #66009-1-Ig, 1:5000) and anti-Rabbit secondary antibody (Proteintech, Cat: #SA00001-2, 1:5000).

### 2.20 Immunohistochemical staining

The Human Protein Atlas (HPA; accessible at https://www.proteinatlas.org/) represents a publicly accessible compendium that systematically documents the spatial distribution and abundance of human proteins throughout diverse cellular, tissue, and organ contexts by integrating multiple experimental methodologies, including immunohistochemistry, microscopy, and transcriptomics. Leveraging this platform, we evaluated immunohistochemical staining characteristics of candidate hub genes in histological sections derived from both normal mammary tissue and breast carcinoma cases.

### 2.21 Cell proliferation and colony formation assays

Cell proliferative capacity was determined via CCK-8 assay (Vazyme, Nanjing, China) using 2×10³ cells/well in 96-well format. The protocol involved 2-hour incubation with CCK-8 reagent at 37 °C in dark conditions, followed by daily absorbance measurements at 450 nm (days 1-4) on a Thermo plate reader. Clonogenic potential was assessed by plating 1, 000 cells/well in 6-well plates with 14-day culture until colony formation. The processing included PBS washing, 4% PFA fixation (15 min), crystal violet staining (20 min; Solarbio, China), and final colony enumeration after air-drying.

### 2.22 Transwell assay

Transwell assays were conducted for cell migration and invasion evaluation. We plated 2×10^4^ treated cells in 200 μl of serum-free medium into the upper chamber of Transwell inserts. The lower chamber contained 600 μl of medium supplemented with 10% serum. After the experiment, we fixed the cells with 4% PFA, stained them with 0.1% crystal violet (Solarbio, China), and performed cell counting under a light microscope.

### 2.23 Statistical analysis

Statistical computations were performed in R v4.2.3. Categorical variable analysis used Pearson’s chi-square test, with continuous variables evaluated by Wilcoxon rank-sum methodology. Differential expression significance thresholds incorporated Benjamini-Hochberg FDR correction. Time-to-event data were analyzed through Kaplan-Meier survival curves with Mantel-Cox proportional hazards testing via the “survival” package. Independent prognostic factors were identified through both univariable and multivariable Cox proportional hazards regression analyses. Predictive accuracy of the model was evaluated by ROC curve profiling, with the AUC quantified using the “timeROC” R package. Associations between risk scores and immune cell infiltration levels were examined via Spearman’s rank correlation analysis. For qRT–PCR data, group comparisons were performed using Student’s t-test. A significance threshold of *p* < 0.05 was applied for all tests, unless explicitly stated otherwise.

## 3 Results

### 3.1 Nucleotide metabolism is significantly activated in tumor epithelial cells​

**Figure 1:**
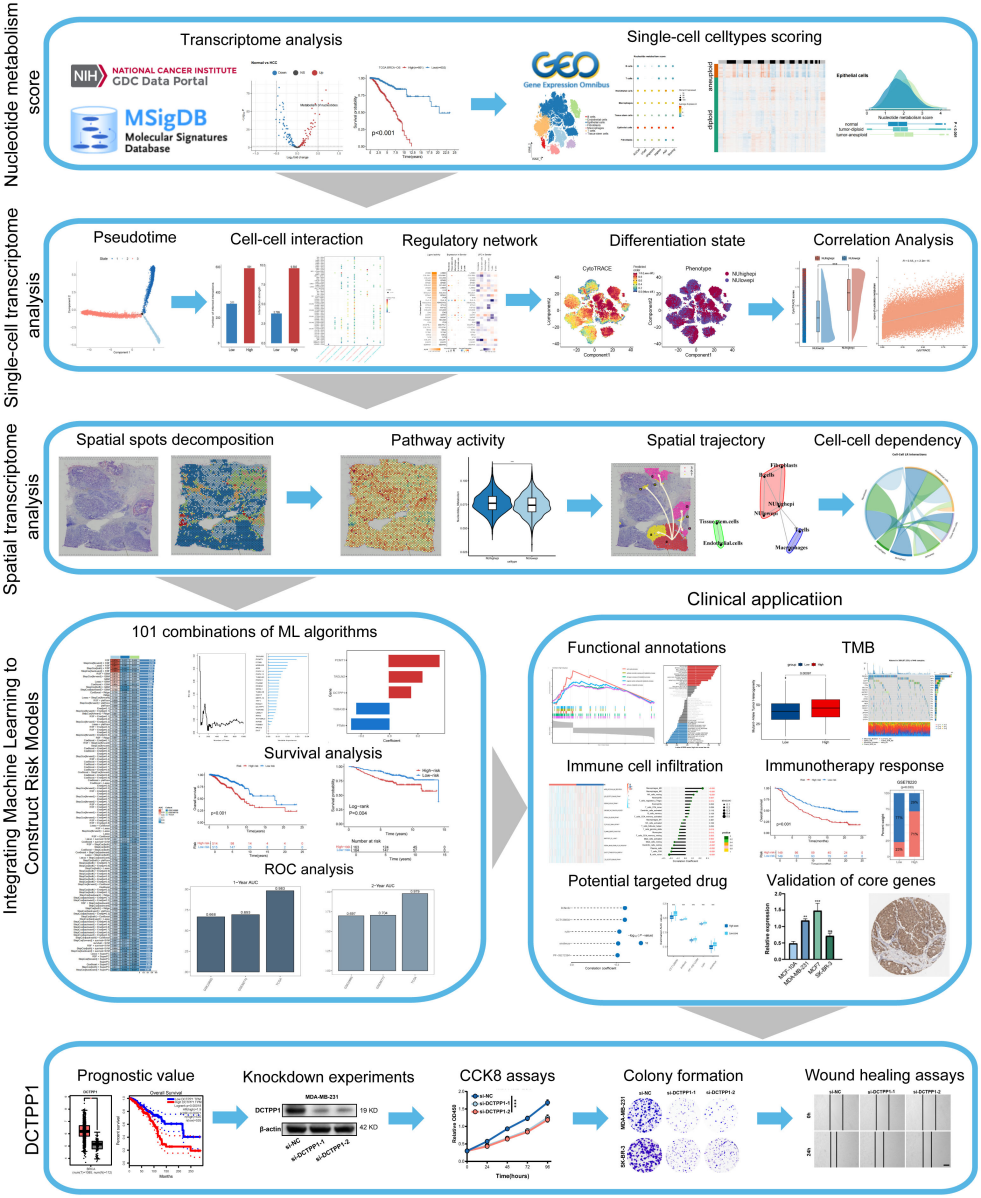
The workflow of the study.

**Figure 2:**
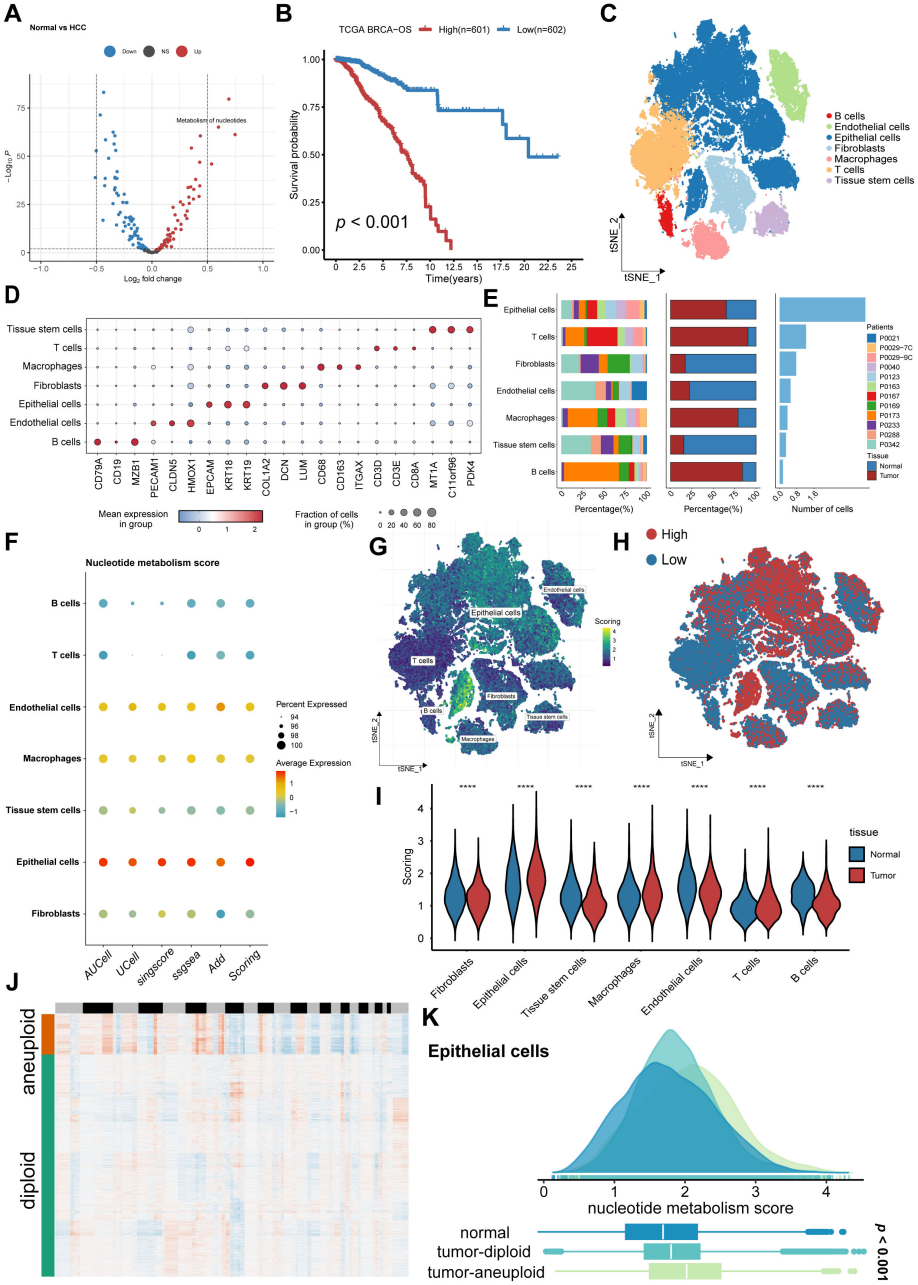
Nucleotide metabolism is increased in tumor epithelial cells: **(A)** Volcano plot displaying differentially metabolism pathways between tumor and normal samples. **(B)** Kaplan–Meier survival analysis of TCGA-BRCA patients stratified by nucleotide metabolism score. **(C)** The tSNE figure with annotations designates distinct cell types with corresponding colors. **(D)** Bubble plot of relative expression of marker genes for each cell type. **(E)** The proportion of each single cells in normal and tumor groups. **(F)** Five scoring methods demonstrate specific enrichment of nucleotide metabolism score in epithelial cells. **(G, H)** Spatial distribution of metabolic activity within tumor sections. **(I)** Violin plots comparing nucleotide metabolism scores among cell subtypes. **(J)** CopyKat algorithm analyzed the distribution of diploid and aneuploid cells. **(K)** Comparison of nucleotide metabolism scores between normal, tumor-aneuploid and tumor-diploid epithelial cells. ****p < 0.0001.

The process of our investigation is illustrated in **Figure 1** comprehensive profiling of metabolic pathway activities in bulk tissues was conducted by leveraging the TCGA-BRCA dataset, which demonstrated a marked enrichment of nucleotide metabolism in tumor specimens relative to normal tissue counterparts (**Figure 2A**, **Supplementary Table 4**). Additionally, **Figure 2B** demonstrates that patients with higher expression levels of nucleotide metabolism-related genes had poorer prognosis (*p* < 0.001). To further elucidate the role of nucleotide metabolism in breast cancer, cell type annotation was performed on the GSE161529 dataset using the CellMarker database and SingleR package, leading to the identification of seven major cell types: T cells, B cells, macrophages, tissue stem cells, fibroblasts, endothelial cells, and epithelial cells (**Figure 2C**). The distinctive cellular markers for these major cell types were visualized using a bubble plot (**Figure 2D**). Patient-specific heterogeneity in cellular composition and abundance across samples was summarized in **Figure 2E**. A notable enrichment of immune cells, particularly T lymphocytes, B cells, and macrophages, was observed within tumor tissues. We subsequently employed five established single cell scoring algorithms to quantify nucleotide metabolic activity. Scores from each method were averaged to generate a unified composite metric termed “Scoring”. The results indicated that epithelial cells had the highest nucleotide metabolism scores, followed by endothelial cells (**Figure 2F**). We further analyzed nucleotide metabolism scores across different cell subpopulations and visualized their distribution at the single-cell level (**Figures 2G, H**). Moreover, epithelial cells in tumor tissues exhibited markedly elevated nucleotide metabolic activity relative to their normal counterparts (**Figure 2I**). We then used CopyKAT software to analyze CNV in all epithelial cells from both tumor and normal tissues, with endothelial cells serving as a reference for diploid cells. The analysis revealed distinct CNV changes in malignant cells, while other epithelial cells tended to remain diploid. Aneuploidy, as predicted by CopyKAT, was predominantly observed in malignant cells, while other epithelial cells were more likely to be diploid (**Figure 2J**). Additionally, nucleotide metabolism scores showed significant variation among normal, tumor-diploid, and tumor-aneuploid cell populations (**Figure 2K**). Based on these metabolic scores, malignant cells were stratified into two distinct clusters: a high nucleotide metabolism group (NUhighepi) and a low nucleotide metabolism group (NUlowepi). This classification enabled further investigation into the biological functions of nucleotide metabolism within malignant epithelial cells.

### 3.2 Elucidating the role of nucleotide metabolism heterogeneity scRNA-seq data

**Figure 3:**
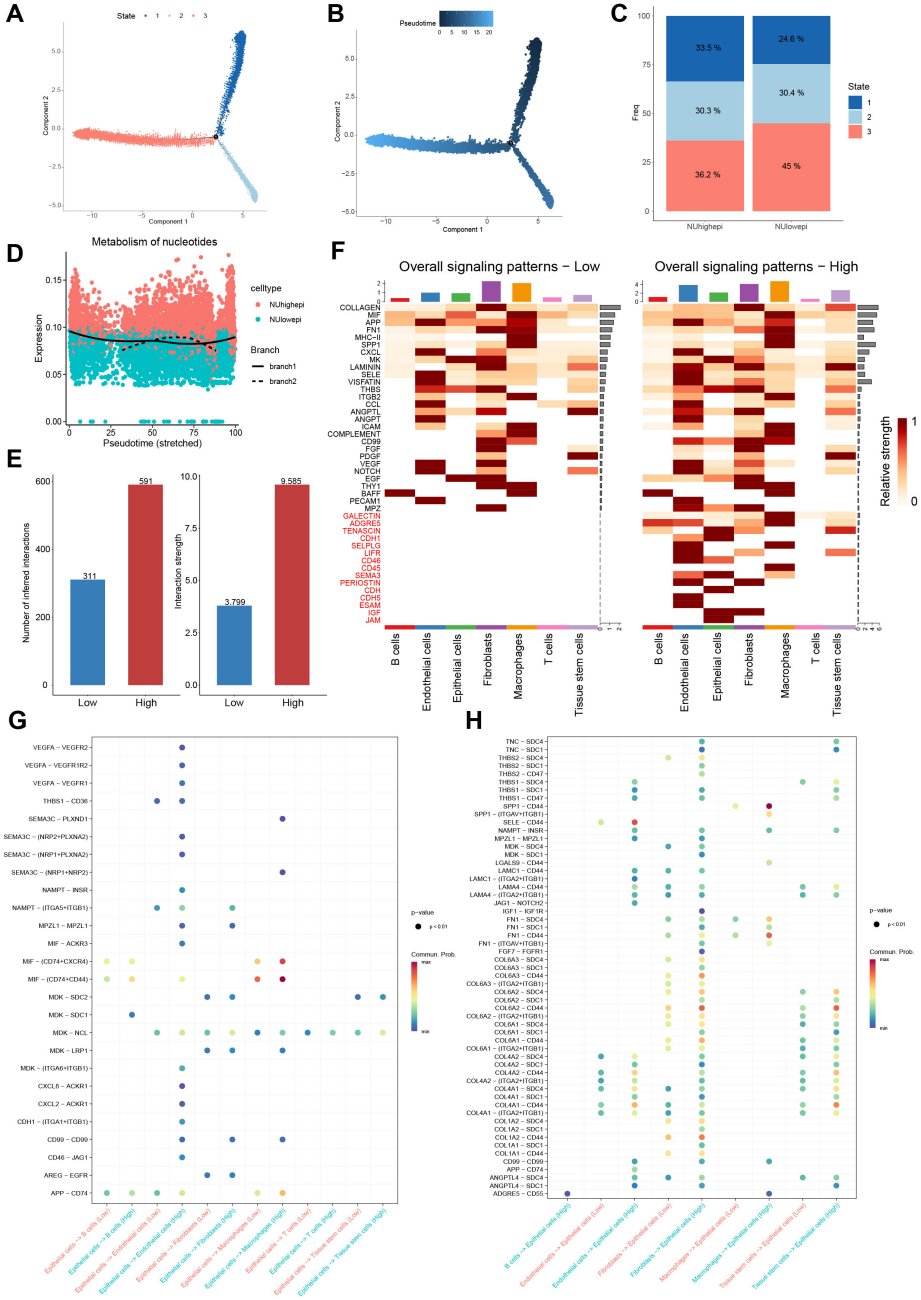
Pseudotime and cell communication analyses.​​ **(A)** Pseudotime analysis of epithelial cells. **(B)** Differentiation trajectories of epithelial cells revealed by Pseudotime analysis. **(C)** Barplot showed pseudotime states of high and low nucleotide metabolism score epithelial cells. **(D)** Expression trends of nucleotide metabolism score during epithelial cells differentiation process. **(E)** Heatmap showing differential cell communication heatmap between high and low nucleotide metabolism score epithelial cells. **(F)** Bubble Chart depicting epithelial cells as ligands in cell communication. **(G)** Bubble Chart depicting epithelial cells as ligands in cell communication. **(H)** Bubble chart illustrating epithelial cells as receptors in cell communication.

Pseudotemporal ordering via Monocle2 demonstrated that NUhighepi populations localize to the origin of the developmental trajectory and persist throughout epithelial differentiation, whereas NUlowepi cells reside predominantly at terminal states (**Figures 3A-C**). This distribution pattern suggests that NUhighepi cells possess higher stemness properties, highlighting the critical role of nucleotide metabolism in tumorigenesis and cancer progression. Furthermore, assessment of nucleotide metabolism activity using a gene signature based scoring system showed an initial increase during early development, followed by a gradual decrease upon cellular maturation (**Figures 3D**). Based on their nucleotide metabolism activity levels, cells were categorized into high score and low score groups. Cell communication analysis revealed that high score cells participated in more substantial signaling interactions compared to low score cells (**Figure 3E**) indicating more active metabolic dynamics within the microenvironment. Similarly, high score epithelial cells demonstrated stronger communicative interactions with other cell types than their low score counterparts (**Figure 3F**). For additional evaluation of the communication differences between NUhighepi and NUlowepi, the expression of receptors and ligands was assessed. Analysis revealed a notable increase in potential ligand-receptor interactions between NUhighepi cells and other cell types relative to NUlowepi populations (**Figures 3G, H**). These findings indicate that NUhighepi cells exhibit enhanced intercellular communication capacity and initiate a broader range of oncogenic and metabolic signaling pathways.

### 3.3 Cell communication analysis of nucleotide metabolism at the single-cell level

**Figure 4:**
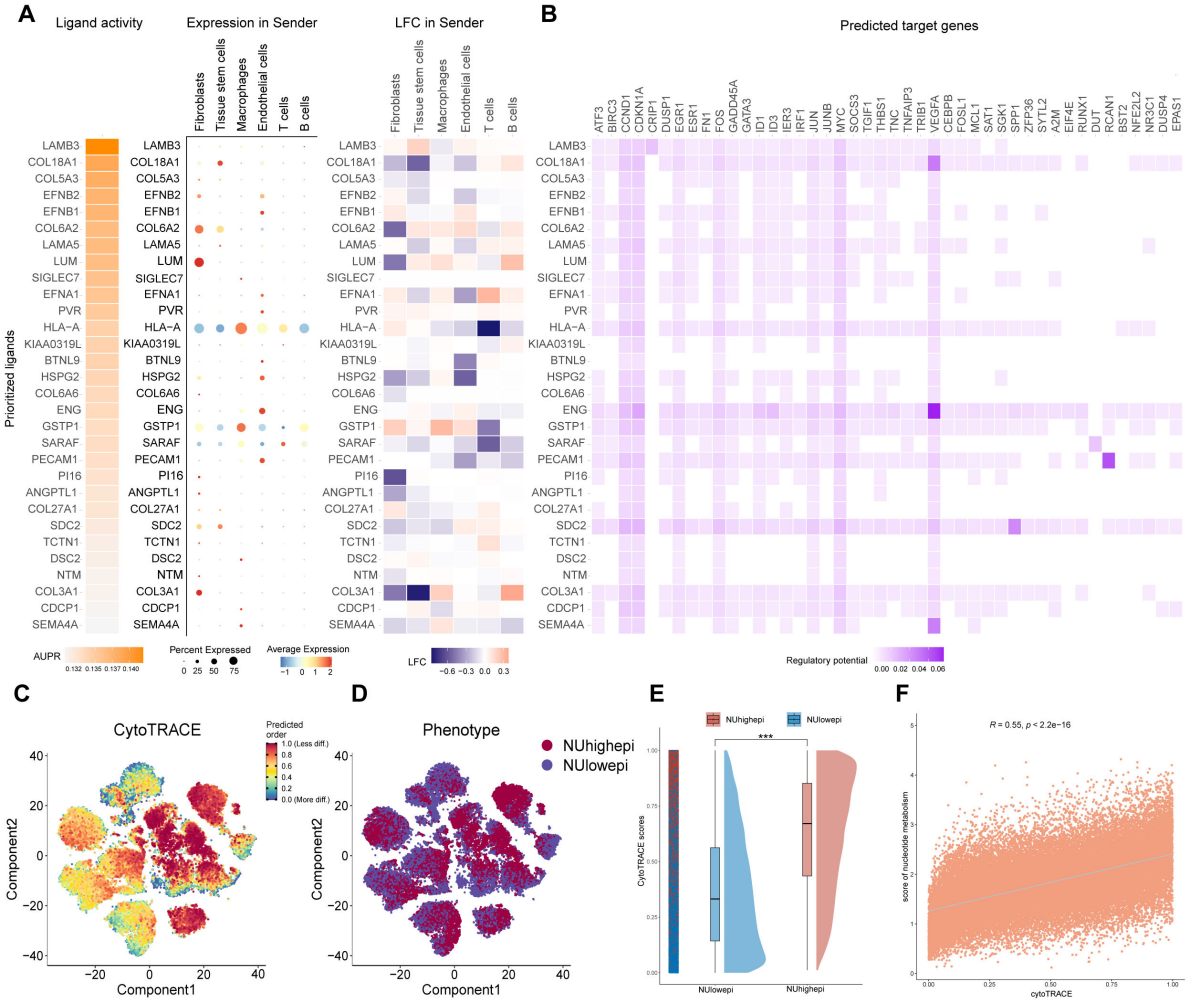
Systematic analysis of ligand-receptor interactions and cellular differentiation states across tumor microenvironment cell types. **(A)** Visualization of the expression of top-predicted ligands and their target genes in a combined heatmap. **(B)** Differential ligands and their target genes between high and low nucleotide metabolism score expression. **(C)** CytoTRACE scatter plot analyzing cellular differentiation states. **(D)** Distribution of NUhighepi and NUlowepi on a TSNE plot. **(E)** Raincloud plot of CytoTRACE scores by NU group in scRNA-seq data. The center of the box plot are median values, the bounds of the box are 25% and 75% quantiles. **(F)** Scatter plot of the correlation between CytoTRACE score and Nucleotide metabolism score. ***p < 0.001.

To delineate intercellular communication networks between epithelial cells and adjacent stromal components, we systematically analyzed ligand-receptor pairing dynamics by examining expression patterns and downstream signaling targets. Our investigation revealed pronounced ligand activity for LAMB3, COL18A1, and COL6A2 within the stromal microenvironment (**Figures 4A, B**). CytoTRACE-based computational assessment demonstrated substantially elevated tumor stemness properties in NUhighepi populations relative to NUlowepi counterparts (**Figures 4C–E**). A positive correlation was observed between nucleotide metabolism enrichment scores and CytoTRACE-derived stemness indices (**Figure 4F**). Pseudotemporal trajectory reconstruction and stemness evaluation supported the notion that NUhighepi cells exhibit multipotent differentiation capacity toward diverse tumor epithelial lineages, indicating heightened plasticity. Furthermore, nucleotide metabolism activity showed significant positive association with genetic markers of cellular differentiation and evolutionary progression, suggesting its promotive role in tumor initiation and advancement.

### 3.4 Spatial transcriptomics to identify nucleotide metabolism in BC

**Figure 5:**
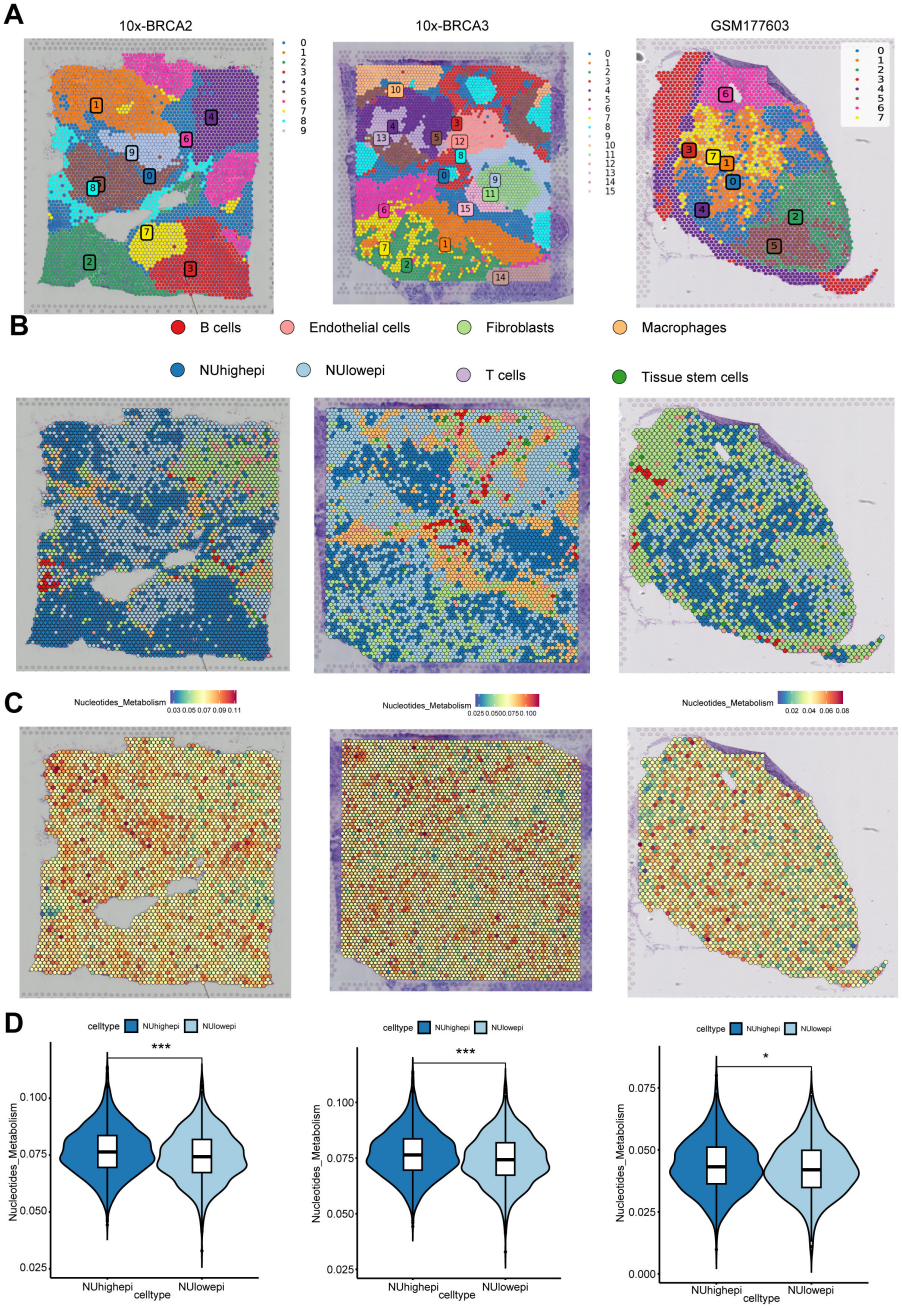
Spatial characterization of nucleotide metabolism heterogeneity in breast cancer tissues. **(A)** Distinct cell sub-populations were identified through the joint annotation of spatial transcriptome sections(10xBRCA2, 10xBRCA3, and GSM6177603) and single-cell data. **(B)** Cell types of each spatial spot revealed by RCTD. **(C)** Featureplot of Nucleotide metabolism in spatial organization. **(D)** Violin plots with boxplot overlays comparing nucleotide metabolism levels between NUhighepi and NUlowepi cells populations across three independent samples. *** *p* < 0.001, * *p* < 0.05.

As described earlier, BC microenvironment undergoes significant modifications in intercellular communication and differentiation states under conditions of nucleotide metabolic dysregulation. To accurately characterize these alterations, spatial transcriptomic profiling of tissue sections was performed. This study specifically analyzed spatial transcriptomics data obtained from three patients enrolled in previously published BC investigations (BRCA2, BRCA3, and GSM6177603). Spatial profiling of these cohorts revealed the organizational patterns of distinct cell populations within tissue sections (**Figure 5A**). Computational deconvolution of scRNA-seq data enabled precise mapping of cellular constituents across the tumor mass and leading-edge regions. Results indicated confined localization of NUhighepi subsets to tumor nests and adjacent peritumoral areas, contrasting with the diffuse spatial patterning observed for NUlowepi populations (**Figure 5B**). These observations imply that elevated nucleotide metabolism might represent a potential therapeutic target for modulating this phenotypic characteristic. Furthermore, significant enrichment of nucleotide metabolic activity was detected within tumor areas (**Figure 5C**). Subsequently, we evaluated the nucleotide metabolic scores between NUhighepi and NUlowepi cells across these three spatial transcriptomic datasets, which yielded results consistent with the prior single-cell data, demonstrating that NUhighepi cells exhibited significantly higher nucleotide metabolic activity compared to NUlowepi cells (**Figure 5D**).

### 3.5 Leveraging multi-modal genomics for deconvolution and cell interaction analysis​

**Figure 6:**
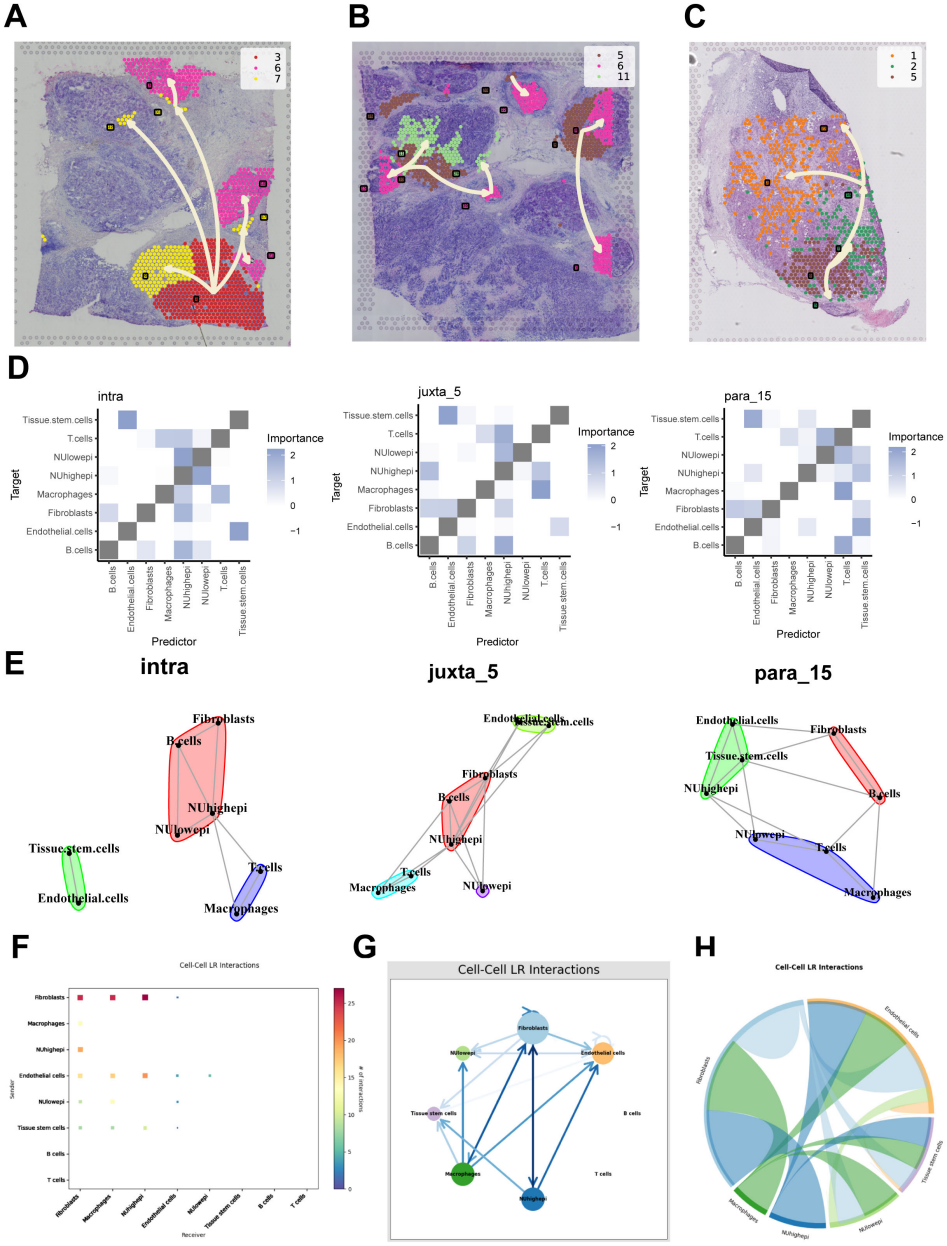
Spatial organization and cell-cell communication networks in the tumor microenvironment. **(A–C)** The developmental trajectories of cell sub-populations from a spatial perspective are investigated. **(D, E)** Heatmap and network diagrams displaying cell–cell dependency analysis in the colocated, neighboring, and extended neighboring (15-point) regions of the spatial transcriptomics data. **(F)** The interaction heatmap visualized the intensity of intercellular interactions mediated by the ligand-receptor pairs. **(G)** The spatial cell communication network diagram illustrates that NUhighepi exhibit a higher intensity of cell communication with other cells. **(H)** Circos plot summarizing cell-type-specific interaction patterns.

Spatial analysis of cellular developmental trajectories revealed that NUhighepi cells, characterized by enhanced nucleotide metabolism, demonstrated differentiation potential toward NUlowepi regions (**Figures 6A–C**). Deconvolution of three tumor samples enabled the application of the MISTy framework (Multiview Intercellular SpaTial modeling) for interrogating cell–cell interactions within spatial transcriptomic data. MISTy offers an interpretable machine learning structure tailored to single-cell, highly multiplexed, and spatially resolved datasets, facilitating deeper understanding of inter- and intracellular regulatory mechanisms. This framework accommodates user-defined views representing distinct spatial contexts, such as intracellular signaling, paracrine activity, and type-specific cellular relationships. Evaluation of view-specific contributions through bar plot visualization identified intraview and paraview15 as the most influential across all samples (**Figures 6D, E**). These outcomes align with prior observations, notably the strong association between epithelial cells exhibiting high nucleotide metabolism and fibroblast prevalence. Subsequently, we analyzed 10x-BRCA2 slide using the “Stlearn” package in Python, indicating robust interactions linking NUhighepi cells to fibroblasts and endothelial cells at the spatial level, whereas NUlowepi cells displayed comparatively limited connectivity with surrounding cell types (**Figures 6F–H**). Spatial profiling identified the top 10 most significant ligand-receptor pairs within tissue domains, as visualized in **Supplementary Figure S1A**. Quantitative analysis of the LGALS1/COL1A2/COL1A1-ITGB1 interaction revealed substantial spatial enrichment and statistical significance (**Supplementary Figure S1B**). The predominant localization of these interactions at tumor cell boundaries indicates their potential role in mediating tumor-stroma crosstalk (**Supplementary Figure S1C**). Further visualization of interaction intensities in **Supplementary Figure S1D** highlighted enhanced communication between NUhighepi cells and both fibroblast and endothelial populations. The results in **Supplementary Figures S1E, F** further confirmed this observation and unveiled the complexity of this spatial cell-cell communication pattern. Our results indicate that nucleotide metabolism disruption enables NUhighepi to orchestrate pathological crosstalk between fibroblasts and endothelial cells, primarily through promoting immunosuppressive microenvironment programming and compromising immunotherapy effectiveness.

### 3.6 Development of a prognostic signature using an integrated machine learning framework

**Figure 7:**
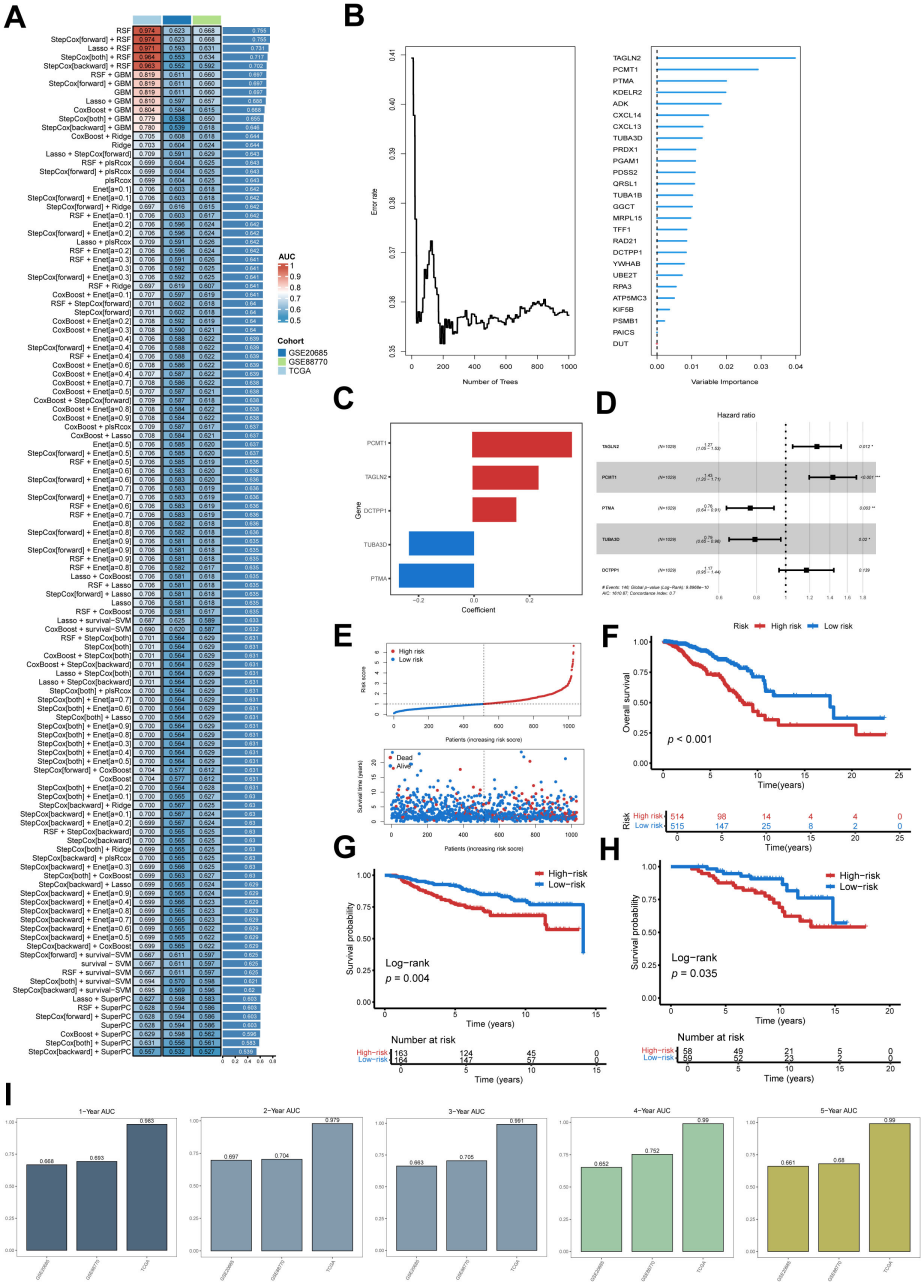
Development and validation of a prognostic model based on multi-algorithm integration and feature selection. **(A)** A total of 101 kinds of prediction models via a tenfold cross-validation framework and further calculated the C index of each model across all validation datasets. **(B)** Random Survival Forest (RSF) model characteristics. **(C)** Regression coefficients of 5 genes obtained in stepwise Cox regression. **(D)** Forest plots showing the results of stepwise Cox regression. **(E)** The distribution of the risk score and overall survival status of patients in the TCGA-BRCA cohort. **(F-H)** Kaplan–Meier curves of OS according to the NMRS in the TCGA, GSE20685, and GSE88770 datasets. **(I)** Predicting patient survival at 1, 2, 3, 4, and 5 years using the NMRS.

To evaluate the potential universality of the NUhighepi subpopulation across cancer types, we analyzed single-cell data from non-small cell lung cancer (NSCLC), ovarian cancer (OV), liver hepatocellular carcinoma (LIHC), and lung squamous cell carcinoma (LSCC) available in the TISCH2 database. Dimensionality reduction and clustering analyses were performed on these datasets (**Supplementary Figures S2A, B**). Using the FindMarkers algorithm applied to scRNA-seq data (GSE161529), we identified 168 genes significantly associated with the NUhighepi subpopulation. The NUhighepi signature scores were subsequently calculated across all samples using ssGSEA. Our results demonstrated that malignant epithelial cells consistently exhibited elevated NUhighepi scores compared to other cell types (**Supplementary Figure S2C**), supporting the broad prevalence of this subpopulation across diverse malignancies and implicating its transcriptional activity in tumor progression. Next, a total of 37 DEGs demonstrating prognostic relevance were subsequently selected through univariate Cox regression for further analysis (**Supplementary Table 5**). To establish a robust nucleotide metabolism-related signature (NMRS), we integrated 101 distinct machine learning approaches to evaluate these 37 candidate genes derived from Cox regression. These predictive models were applied to the TCGA dataset, and the concordance index (C-index) was computed across two independent validation cohorts. Integration of RSF algorithms produced the most performant model, attaining an average C-index of 0.755 (**Figure 7A**). The RSF algorithm further identified 20 hub genes critical to the signature (**Figure 7B**). Patient-specific risk scores were generated by weighting the expression levels of five pivotal genes according to their Cox regression coefficients (**Figures 7C, D**). Based on median risk score stratification, patients were categorized into high NMRS and low NMRS subgroups. Elevated risk scores correlated with increased patient mortality (**Figure 7E**). Survival analyses conducted across training and internal validation sets revealed significantly poorer overall survival in high NMRS patients compared to low NMRS individuals (**Figures 7F–H**). ROC analysis demonstrated strong discriminatory performance of the NMRS, with area under the curve (AUC) values for one- to five-year predictions as follows: TCGA-BRCA: 0.983, 0.979, 0.991, 0.99, 0.99; GSE20685: 0.668, 0.697, 0.663, 0.652, 0.661; GSE88770: 0.693, 0.704, 0.705, 0.752, 0.68 (**Figure 7I**). These consistent results underscore the robustness and generalizability of the NMRS across multiple independent cohorts.

### 3.7 Elucidating molecular mechanisms of the NMRS in bulk RNA-Seq

**Figure 8:**
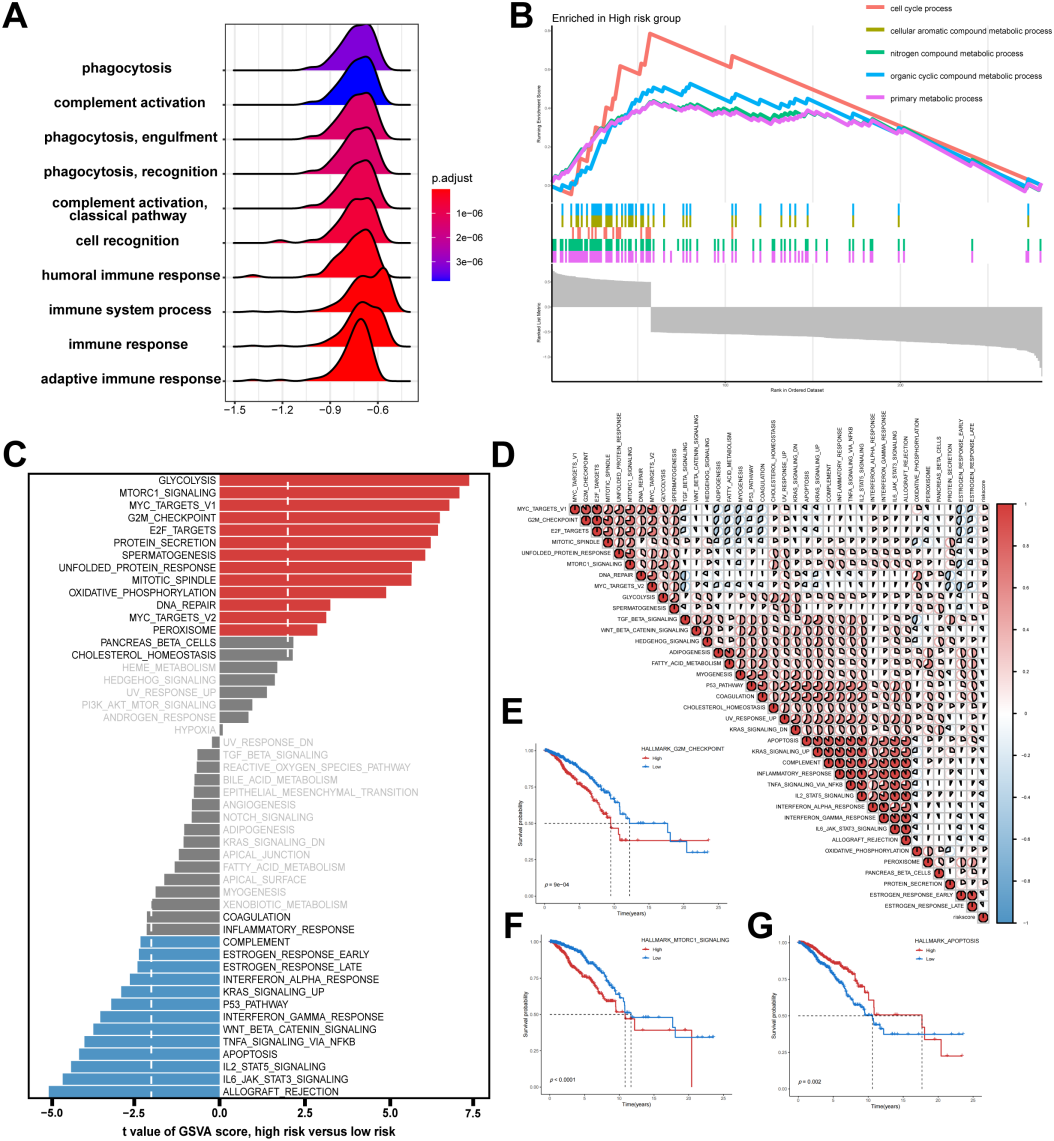
The transcriptome features of patients with various NMRS in BC. **(A)** Ridge plot showing the GO terms enriched in the low NMRS group. **(B)** GO terms enriched in the high NMRS group by GSEA analysis. **(C)** Differences in hallmark pathway activities between the high- and low NMRS groups scored by GSVA. **(D)** Correlation between the risk score and hallmark pathway activities scored by GSVA. **(E-G)** Kaplan–Meier survival plots showing the significant correlations between the OS and GSVA scores of pathways.

To elucidate the molecular basis linking the NMRS with BC prognosis, functional enrichment analysis was conducted. In the GSEA analysis based on the GO gene set, we observed that the low NMRS group was enriched in positive regulation of complement activation, humoral immune response, and adaptive immune response (**Figure 8A**, **Supplementary Table 6****),** whereas the high NMRS group was enriched in cell cycle process, cellular aromatic compound metabolic process, and primary metabolic process (**Figure 8B**). Additionally, the GSVA analysis revealed that the high NMRS group exhibited stronger activity in pathways related to GLYCOLYSIS, MYC_TARGETS_V1, and G2M_CHECKPOINT, whereas the low NMRS group exhibited stronger activity in pathways related to ALLOGRAFT_REJECTION, IL6_JAK_STAT3_SIGNALING, and APOPTOSIS (**Figure 8C**). Correlation analysis of NMRS and hallmark pathway scores provided further support for these observations (**Figure 8D**), demonstrating a close relationship between NMRS and cancer-associated biological processes. To evaluate the prognostic relevance of these Hallmark pathways in BC, Kaplan–Meier (KM) survival analysis was performed. Pathways showing positive correlation with NMRS, including G2M_CHECKPOINT and MYC_TARGETS_V1, were linked to unfavorable clinical outcomes (**Figures 8E, F**). In contrast, pathways exhibiting negative correlation with NMRS, such as APOPTOSIS, correlated with improved survival (**Figure 8G**). These findings suggest that the activation or suppression of these pathways may underlie the differential prognostic outcomes across NMRS-based risk subgroups.

### 3.8 Genomic variation and intratumor heterogeneity across NMRS subgroups​

**Figure 9:**
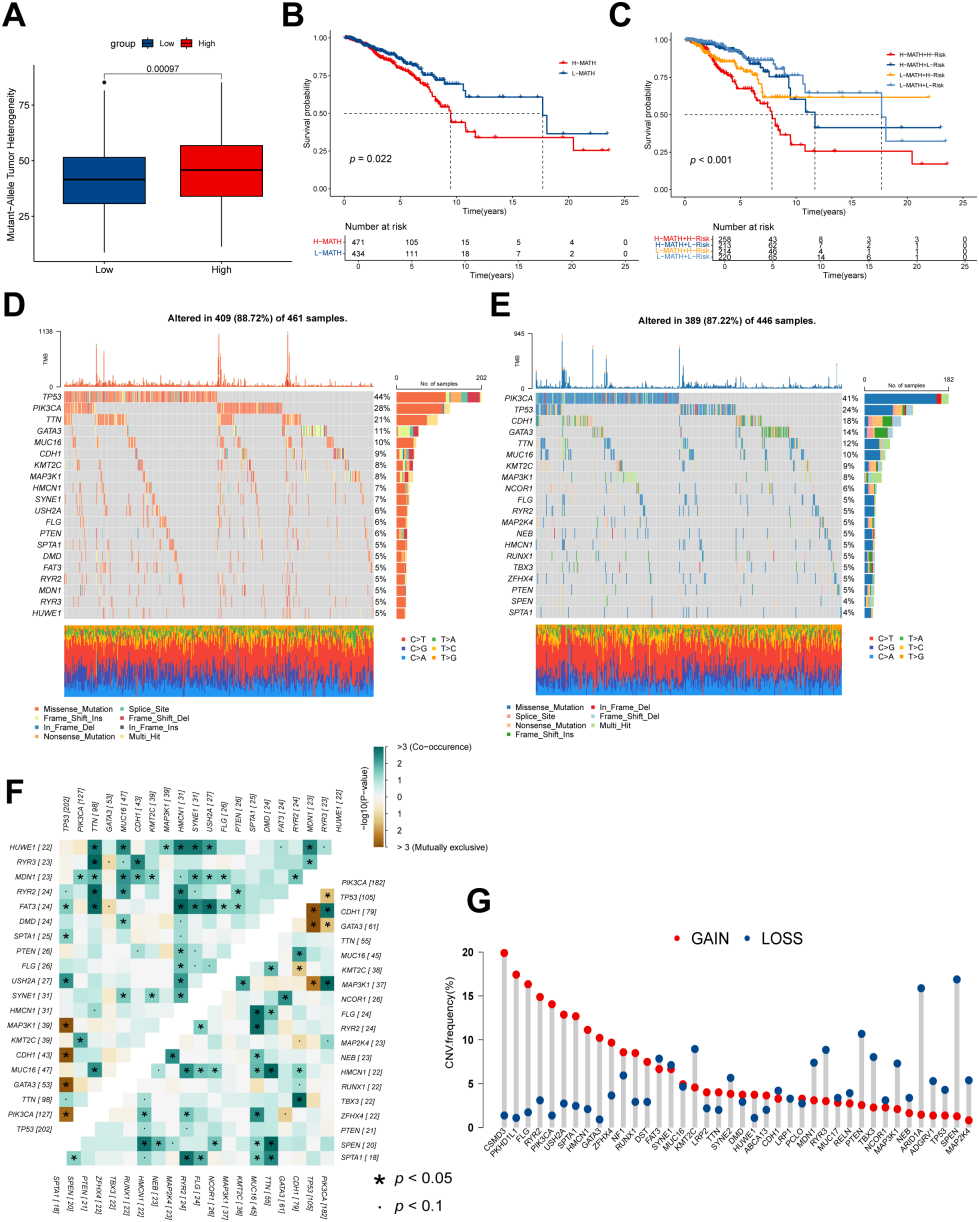
Genetic alterations associated with NMRS between low- and high NMRS groups. **(A)** Boxplot showing the difference in mutant allele tumor heterogeneity (MATH) score between the high- and low NMRS groups. **(B)** Kaplan–Meier curve shows the difference in OS between high- and low-MATH score groups. **(C)** Kaplan–Meier curve analysis for OS by combining the MATH score and the NMRS score. **(D, E)** The waterfall plot of the somatic mutation landscape in high- **(D)** and low NMRS patients **(E)** in the TCGA-BRCA cohort. **(F)** Heatmaps showing the association of co-occurrence and exclusive mutation among the top 25 mutated genes in high- and low NMRS groups. **(G)** Distribution of CNV frequency among DEGs between high- and low NMRS groups. Blue and red, for deletion and amplification, respectively between cells with high- and low NMRS scores.

ITH represents a fundamental genomic feature of cancer, arising from the progressive accumulation of genetic alterations (36). This heterogeneity has been closely linked to advanced disease progression and heightened resistance to chemotherapeutic agents (37). Within this study, ITH was quantitatively assessed in BC patients via the MATH algorithm, wherein elevated MATH scores correspond to increased heterogeneity. Analysis revealed that high NMRS BC patients exhibited significantly higher MATH scores (**Figure 9A**). Further investigation into the prognostic relevance of ITH demonstrated that patients with high MATH scores experienced markedly reduced OS compared to those with lower scores (**Figure 9B**). Integration of ITH with the NMRS classification revealed that the “high NMRS + high MATH” subgroup had a significantly worse prognosis than the “low NMRS + low MATH” subgroup (*p* < 0.001), indicating that combined evaluation using these two markers enhances prognostic accuracy for BC patients (**Figure 9C**). To investigate the differences in genomic mutations between NMRS subgroups, we depicted the mutation landscape between high- and low NMRS groups (**Figures 9D, E**). In the high NMRS cohort, predominant mutations were identified in TP53, PIK3CA, and TTN (**Figure 9D**), whereas the low NMRS cohort exhibited the highest mutation frequencies in PIK3CA, TP53, and CDH1 (**Figure 9E**). The high NMRS subgroup displayed an elevated overall burden of somatic mutations relative to the low NMRS subgroup. These divergent mutational profiles may underlie the differential prognostic outcomes observed across NMRS-based classifications. Further evaluation of co-occurrence and mutual exclusivity patterns among the top 25 most frequently mutated genes indicated more prevalent co-mutation events within the high NMRS subgroup (**Figure 9F**). CNV analysis was subsequently performed for the 30 genes demonstrating the most pronounced differential expression between subgroups (**Figure 9G**). Recurrent CNV gains were observed in CSMD3, PKHD1L1, and FLG, whereas SPEN, ARID1A, and PTEN were characterized by frequent CNV losses.

### 3.9 Association between the NMRS and immune microenvironment landscapes​

**Figure 10:**
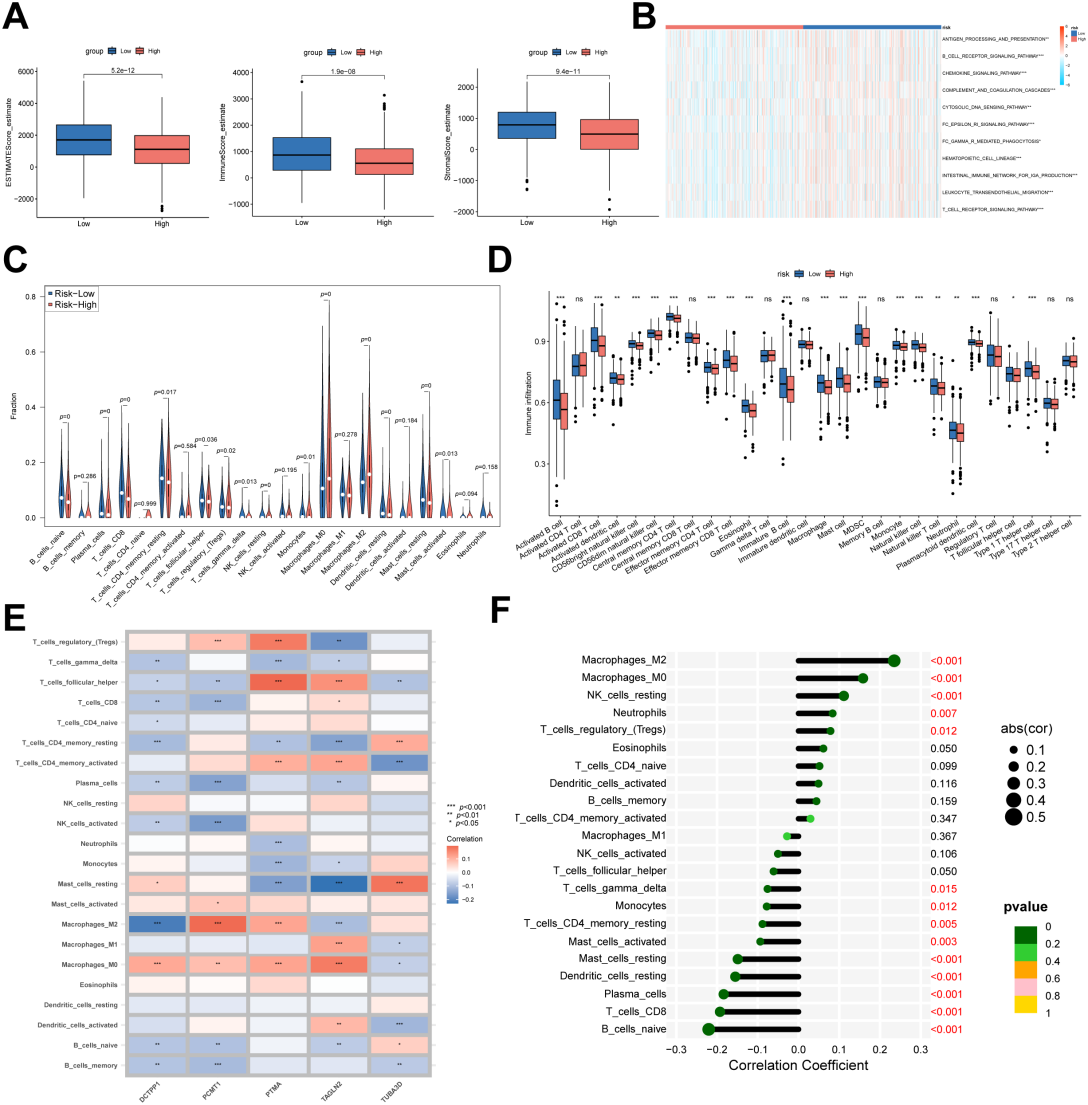
The immune landscape associated with NMRS in BC. **(A)** The immune score, the ESTIMATE score, and the tumor purity were applied to quantify the different immune statuses between the high- and low NMRS groups. **(B)** Immune-related pathways’ activity showing a significant difference between high- and low NMRS groups. **(C, D)** The abundance of each TME-infiltrated cell type between high- and low NMRS groups, quantified by the CIBESORT algorithm **(C)** and the ssGSEA algorithm **(D, E)** The association between TME-infiltrated cells and genes built-in NMRS. **(F)** Correlation analysis between TME-infiltrated cells and NMRS. ***p < 0.001, **p < 0.01, *p < 0.05, ns *p* > 0.05.

To characterize the immune infiltration landscape in breast cancer samples, we utilized the ESTIMATE algorithm to compute stromal, immune, and composite ESTIMATE scores across NMRS-defined risk subgroups. Analysis revealed that the high NMRS subgroup displayed markedly reduced immune, stromal, and ESTIMATE scores compared to the low NMRS subgroup (**Figure 10A**). Pathway activity profiling via ssGSEA further demonstrated significantly enhanced activation of antigen presentation, chemokine signaling, and T-cell receptor pathways in the low NMRS subgroup (**Figure 10B**). To delineate differences in immune cell composition, we employed the CIBERSORT algorithm to quantify infiltrating immune cell abundances in each sample (**Figure 10C**). Results indicated elevated levels of CD8^+^ T cells, activated CD4^+^ memory T cells, and follicular helper T cells in the low NMRS subgroup. In contrast, the high NMRS subgroup exhibited greater abundances of non-cytolytic cell types, including resting CD4^+^ memory T cells, M2 macrophages, and resting mast cells. Consistent outcomes were observed upon validation using the ssGSEA algorithm (**Figure 10D**). Moreover, we found that five genes within the NMRS were highly correlated with tumor-infiltrating immune cells, where TAGLN2 showed a positive correlation with T cell CD8 and T cell CD4 memory activated, and PTMA and PCMT1 were positively correlated with macrophage M2 and Tregs. Subsequently, Spearman’s correlation analysis was utilized to identify immune cell subtypes showing statistically significant associations with NMRS (**Figure 10E**). This approach led to the recognition of 14 distinct immune cell categories that met the threshold of statistical significance (*p* < 0.05; **Figure 10F**). These results imply that NMRS could potentially serve as an informative biomarker for evaluating immune cell infiltration within the tumor microenvironment of BC patients.

### 3.10 Association of the NMRS with anti-tumor immunity and immunotherapeutic efficacy​

**Figure 11:**
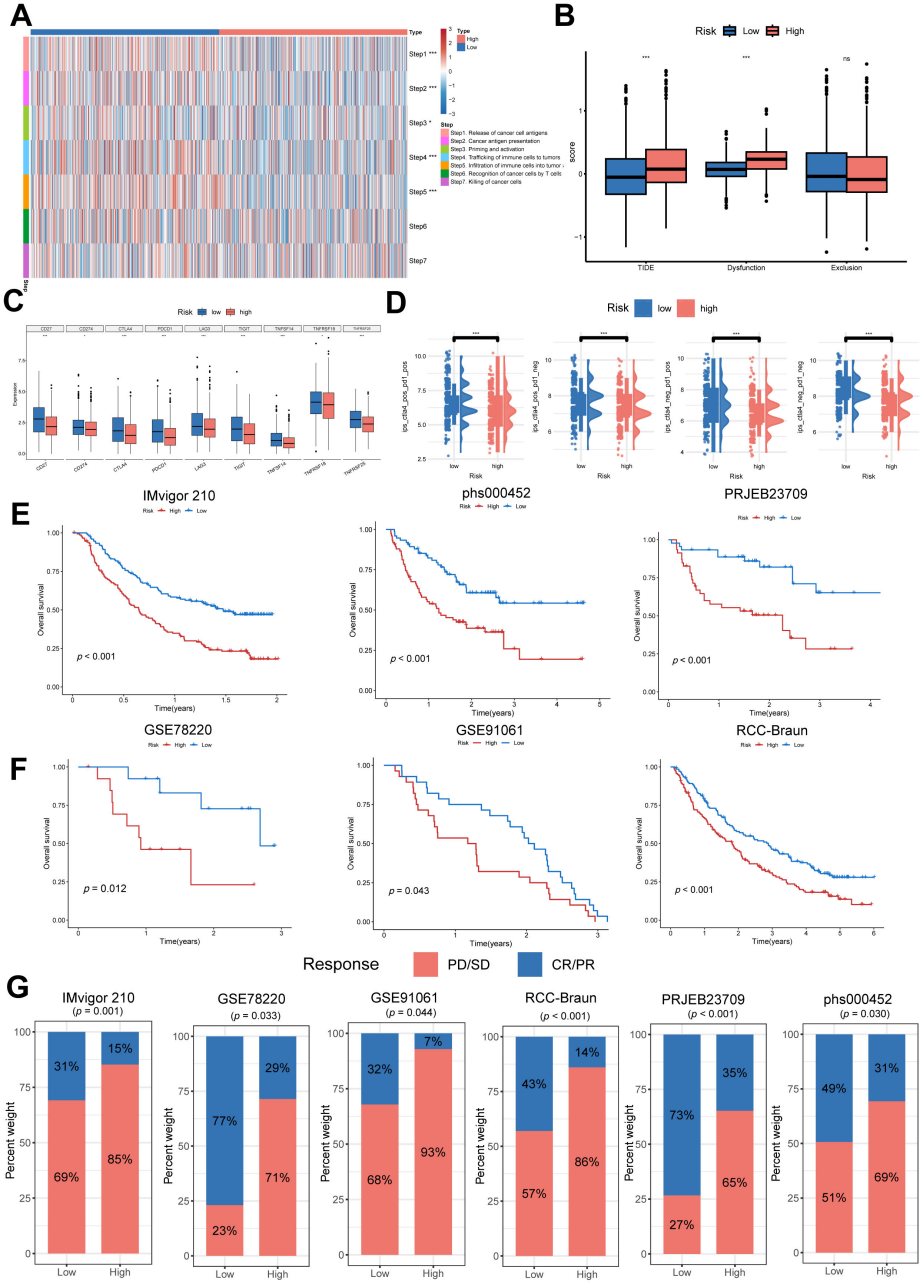
The relationship between the NMRS and immunotherapy response. **(A)** Heatmap showing the difference in the seven-step anti-cancer immunity cycle activity between high- and low NMRS groups. **(B)** The boxplot demonstrating the difference in the TIDE score of NMRS subgroups. **(C)** The expression of immune checkpoints in high- and low NMRS groups. **(D)** The IPS score between high- and low NMRS groups. **(E)** Kaplan-Meier estimates of overall survival of patients treated with immunotherapy in metastatic urothelial carcinoma (IMvigor210), SKCM (phs000452.v3.p1) and Melanoma (PRJEB23709). **(F)** Kaplan-Meier estimates of overall survival of patients treated with immunotherapy in Melanoma (GSE78220), SKCM (GSE91061) and KIRC (KIRC David Braun et.al) **(G)** The response to immunotherapy in different NMRS group patients. Response means CR/PR and Non-response means PD/SD (CR, complete response; PD, progressive disease; PR, partly response; SD, stable disease). *** *p* < 0.001, * *p* < 0.05, ns *p* > 0.05.

Owing to the intricate interplay between immune processes and the tumor microenvironment, merely quantifying immune cell infiltration proves insufficient for characterizing states of immune activation and exhaustion. A more comprehensive assessment can be achieved by evaluating the functional activity at each step of the anti-cancer immunity cycle, thereby refining prognostic insights and therapeutic guidance for immunotherapy (38). As illustrated in **Figure 11A**, pronounced disparities were identified across multiple steps (1–5) of the anti-cancer immunity cycle among NMRS-based risk subgroups. The low NMRS subgroup exhibited heightened activity in immune priming and activation (Step 3), immune cell trafficking toward tumor sites (Step 4), and T cell-mediated cancer cell recognition (Step 6), collectively implying superior anti-tumor immune functionality. To gauge clinical implications for immunotherapy responsiveness, the TIDE algorithm was employed. Elevated TIDE scores are indicative of a heightened propensity for immune escape, corresponding to reduced benefits from immune checkpoint inhibition. Within the TCGA cohort, the high NMRS subgroup demonstrated significantly higher TIDE scores relative to the low NMRS subgroup (**Figure 11B**). Consistent with previous findings, increased expression of immune checkpoint molecules—including PDCD1 (PD-1), CTLA-4, TIGIT, LAG3, and TNFRSF25—was correlated with improved response to immune checkpoint inhibitor (ICI) therapy (39). Accordingly, expression levels of these immunoregulatory molecules were compared across NMRS subgroups, revealing significantly elevated expression in the low NMRS subgroup (**Figure 11C**). To further verify our results, we analyzed IPS scores obtained from the TCIA database. Higher IPS scores predict a better response to ICI therapy, which includes PD-1 inhibitor and CTLA4 inhibitor therapy classified into four categories: (1) ips_ctla4_pos_pd1_pos (CTLA4 + /PD1 + treatment), (2) ips_ctla4_pos_pd1_neg (CTLA4 + /PD1- treatment), (3) ips_ctla4_neg_pd1_pos (CTLA4-/PD1 + treatment), and (4) ips_ctla4_neg_pd1_neg (CTLA4-/PD1- treatment). Analysis of the IPS revealed significantly reduced scores in high NMRS patients across both CTLA4+/PD1+ and CTLA4+/PD1- treatment categories. These findings suggest that elevated NMRS patients exhibit diminished responsiveness to anti-CTLA4 monotherapy, as well as to anti-PD-1 and anti-CTLA4 combination regimens, relative to the favorable NMRS subgroup (**Figure 11D**).

We systematically evaluated the NMRS’s prognostic value in immune checkpoint inhibitor therapy using multi-center datasets targeting PD-1/PD-L1 pathways. Results revealed consistently superior overall survival in low-NMRS patients spanning melanoma (SKCM), urothelial carcinoma (UC), renal cell carcinoma (KIRC) and metastatic urothelial malignancies (**Figures 11E, F**), suggesting that high NMRS levels potentially compromise immunotherapeutic benefits. Treatment response stratification further confirmed significant disparities between NMRS-defined subgroups. Furthermore, the response to PD-1/PD-L1 ICI treatment differed between patients with high and low NMRS group. The pan-cancer analysis revealed that patients with elevated NMRS group exhibited a poor response to ICI treatment, whereas over fifty percent of patients with low NMRS group demonstrated a positive response (**Figure 11G**). Specifically, the high NMRS group primarily exhibited no response (progressive disease or stable disease), whereas the low NMRS group mostly showed a response (complete response or partial response). These collective findings underscore the robustness of NMRS as a predictive biomarker for immunotherapy efficacy and suggest its potential utility in identifying patients most likely to benefit from treatment.

### 3.11 Predicting drug sensitivity with the NMRS and validating gene expression profiles

**Figure 12:**
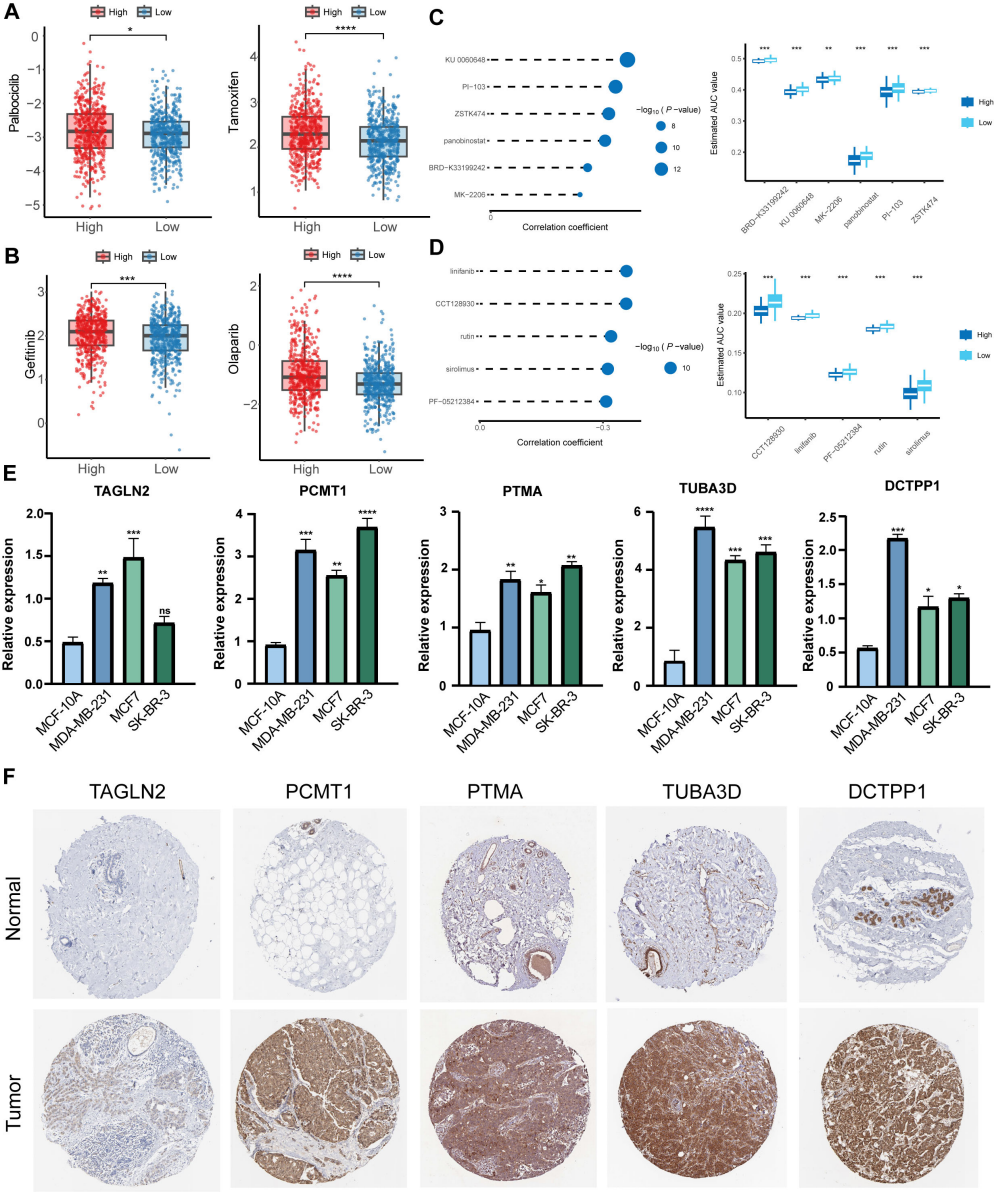
Predicting therapy response with NMRS and validation of the core genes. **(A)** Estimated IC50 values of Tamoxifen and Palbociclib in high- versus low NMRS groups. **(B)** Estimated IC50 values of Olaparib and Gefitinib in high- versus low NMRS groups. **(C, D)** The correlation and differential analysis of drug sensitivity for potential drugs screened from the CTRP and PRISM datasets. **(E)** Validation of the expression of TAGLN2, PCMT1, PTMA, TUBA3D, and DCTPP1 in a normal breast cell line (MCF-10A) and three BC cell lines (MDA-MB-231, MCF7, and SK-BR3) by qRT-PCR. **(F)** Immunohistochemical images showing the protein expression of the five genes in the HPA database. ****p < 0.0001, ***p < 0.001, **p < 0.01, *p < 0.05, ns *p* > 0.05. BC breast cancer, HPA Human Protein Atlas.

We employed drug sensitivity data from the Genomics of Drug Sensitivity in Cancer (GDSC) to compute half-maximal inhibitory concentration (IC50) values for therapeutic agents in breast cancer patients. Comparative assessment of standard endocrine and targeted therapies revealed substantially elevated IC50 values in the high NMRS subgroup relative to the low NMRS cohort (**Figures 12A, B**), highlighting the urgent need for effective treatments targeting NMRS-elevated populations. Subsequent screening of the Cancer Therapeutics Response Portal (CTRP) and PRISM Repurposing datasets identified 356 and 1, 291 compounds respectively after quality control. Differential response analysis between extreme NMRS deciles pinpointed agents with reduced efficacy in high-NMRS groups. Further refinement through Spearman correlation filtering (R < -0.2) yielded 6 CTRP and 5 PRISM compounds exhibiting preferential activity against high-NMRS tumors (**Figures 12C, D**). These compounds exhibit considerable potential as targeted therapeutics to impede carcinogenesis and tumor advancement, presenting opportunities for both interventional and prophylactic applications in breast cancer management. Subsequent investigation quantified the transcriptional profiles of the five-gene NMRS panel across a cellular model system comprising normal mammary epithelium (MCF-10A) and three malignant breast cell lines (MDA-MB-231, MCF7, SK-BR-3) (**Figure 12E**). Analytical results demonstrated substantial elevation in the expression of particular genetic elements. To further validate these observations, immunohistochemistry (IHC) was employed to examine protein expression levels of the five candidate genes in BC and normal control tissues sourced from the HPA database (**Figure 12F**). Consistent with RNA sequencing results, all five genes exhibited elevated expression in tumor tissues compared to normal samples.

### 3.12 DCTPP1 knockdown inhibits the activity of BRCA cells

**Figure 13:**
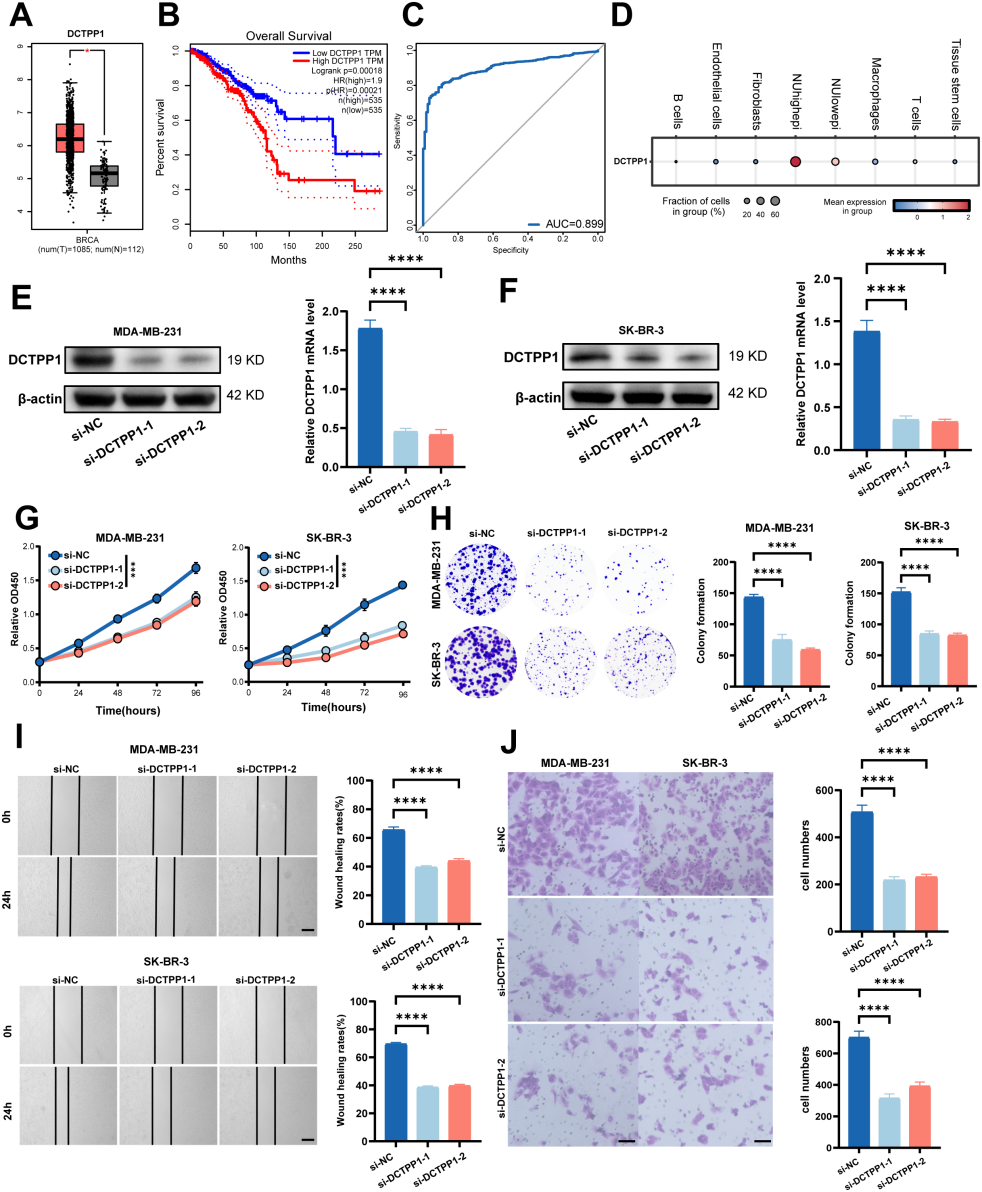
DCTPP1 expression and functional validation in BC **(A)** DCTPP1 mRNA expression was higher in TCGA-BRCA tumor tissues than in normal tissues (*p* < 0.01). **(B)** Overall survival was worse in TCGA-BRCA patients with high DCTPP1 expression (*p* < 0.0001). **(C)** ROC curve analysis of DCTPP1, highlighting its diagnostic potential. **(D)** Single-cell analysis showed (GSE161529) that DCTPP1 expression was significantly higher in NUhighepi cells than in others. **(E, F)** WB and qpcr analysis of the knockdown efficiency of DCTPP1 in MDA-MB-231 **(E)** and SK-BR-3 **(F)** cells and showed that the DCTPP1 gene was successfully knocked down in MDA-MB-231 and SK-BR-3 cell lines. **(G)** CCK-8 showed that the proliferation activity of the cells that knockdown DCTPP1 was dramatically reduced. **(H)** After DCTPP1 knockdown, the cloning ability of MDA-MB-231 and SK-BR-3 cell lines decreased significantly. **(I)** Healing test. After DCTPP1 knockdown, the migration ability of MDA-MB-231 and SK-BR-3 cell lines decreased significantly. **(J)** Transwell assay. After DCTPP1 knockdown, the migration and invasion abilities of MDA-MB-231 and SK-BR-3 cell lines were significantly decreased. ****p < 0.0001, ***p < 0.001, *p < 0.05, ns *p* > 0.05.

To further investigate how NMRGs regulate tumor progression in breast cancer, we focused on DCTPP1, a key gene within the NMRGs. Literature reports indicate that DCTPP1 is a promising target for cancer therapy and prognosis prediction through nucleotide metabolism (40). DCTPP1 expression was significantly elevated in tumor tissues compared to normal breast tissues in TCGA-BRCA cohort, as shown in **Figure 13A**. Kaplan–Meier survival analysis indicates that high DCTPP1 expression is associated with poorer overall survival in BC patients (**Figure 13B**). To evaluate the diagnostic potential of DCTPP1, we performed ROC curve analysis using the TCGA-BRCA cohort dataset. DCTPP1 demonstrated a high AUC value, indicating its potential as a diagnostic biomarker for breast cancer (**Figure 13C**). The expression of DCTPP1 in different breast cancer cell types, shown through bubble plot, reveals that DCTPP1 is primarily expressed in NUhighepi cells (**Figure 13D**). To investigate the functional role of DCTPP1, we performed knockdown experiments targeting DCTPP1 in MDA-MB-231 and SK-BR-3 breast cancer cell lines using siRNA. Western Blot (WB) and Quantitative RT-PCR analysis confirmed successful reduction of DCTPP1 expression in MDA-MB-231 and SK-BR-3 cells (**Figures 13E, F**). We evaluated proliferative effects using CCK-8 assays, observing substantially decreased growth kinetics in DCTPP1-knockout MDA-MB-231 cells compared with controls. SK-BR-3 cells exhibited a similar proliferation deficit pattern (**Figure 13G**). Colony formation analysis showed that DCTPP1 suppression led to fewer and smaller colonies in both BRCA cell types (**Figure 13H****).** In motility assays, we detected significantly reduced migration frequencies in DCTPP1-deficient cells across wound healing and Transwell platforms, with bar chart analysis confirming statistically robust differences (**Figure 13I**). These findings strongly suggest that DCTPP1 knockdown compromises the migration and invasion capabilities of both breast cancer cell models (**Figure 13J**).

## 4 Discussion

Nucleotide metabolism plays a pivotal role in oncogenesis and malignant progression (41). One underlying mechanism may involve the limited capacity of the tumor microenvironment (TME) to supply nucleotides in adequate amounts or optimal proportions, necessitating enhanced metabolic processing of nonessential amino acids, ribose, and one-carbon units by proliferating cells to support biosynthesis (42). Breast cancer (BC) exhibits high incidence and mortality rates worldwide. Consequently, suppressing nucleotide metabolism may represent a promising therapeutic strategy either to directly target cancer cells or to augment the effectiveness of existing anticancer regimens. In this investigation, we employed single-cell RNA sequencing, spatial transcriptomics, bulk RNA-seq, and fundamental experimental approaches to systematically characterize the malignancy-associated properties of nucleotide metabolism with single-cell and spatial resolution.

This research delineated the cellular and spatial immunologic characteristics of nucleotide metabolism in BC through integrated analysis of single-cell RNA sequencing and spatial transcriptomic data. Our findings indicate a marked enhancement of nucleotide metabolic activity within tumor epithelial populations, an observation substantiated by multiple computational scoring methodologies. Furthermore, neoplastic cells exhibiting elevated nucleotide metabolism demonstrated increased capacities for tumor stemness maintenance and proliferative expansion, consistent with previously published reports (8, 9). Of particular note, the cellular state designated as NUhighepi was identified within a terminal differentiation compartment and displayed a pronounced propensity for metastatic dissemination. This research not only uncovers previously unexplored aspects of nucleotide metabolism and its interaction with the immune system but also provides a valuable resource for advancing cancer research.

Emerging evidence highlights that the altered manipulation of nucleotides by cancer cells facilitates their evasion of immune surveillance through various mechanisms (43). Comprehensive characterization of the tumor microenvironment’s spatial architecture was achieved through integrated analysis of scRNA-seq and spatial transcriptomic datasets. We discovered that the LGALS1/COL1A2/COL1A1-ITGB1 axis demonstrated exceptional interaction potency at tumor-stroma interfaces, indicating its function as a critical regulator of intercellular spatial dialogue. Quantitative assessment of communication patterns showed intensified signaling between NUhighepi clusters and stromal components (fibroblasts/endothelial cells), corroborating the premise that these cells orchestrate TME remodeling through dysregulated signaling pathways. Such pathological communication networks likely facilitate tumor advancement by providing a supportive niche for expansion and metastasis. Spatial multi-omics integration thus unveiled both the topographic distribution of NUhighepi populations and their mechanistic involvement in disease progression. Cancer-associated fibroblasts (CAFs) are increasingly recognized as principal contributors to immunosuppression within the tumor microenvironment TME (44). CAFs modulate immune cell infiltration through direct secretion of cytokines, chemokines, and surface proteins, as well as indirect effects via remodeling extracellular matrix (ECM) components that guide immune cell trafficking and positioning. Additional studies indicate tumor endothelial cells (TECs) promote immune tolerance under hypoxic conditions (45) and engage in crosstalk with CAFs through VEGFA signaling (46). In specific malignancies, TECs upregulate immune checkpoint molecules on T cells, thereby inhibiting T cell activation (47). FasL-expressing TECs may diminish CD8^+^ T cell populations while expanding regulatory T cells (Tregs), and mounting evidence correlates hypoxia-mediated TME remodeling with poor clinical outcomes (48). Hypoxia induces resistance to conventional therapies by affecting multiple pathways including apoptosis, autophagy, DNA damage response, mitochondrial function, p53 signaling, and drug efflux mechanisms. These findings suggest synergistic interactions between these stromal components and NUhighepi cells in coordinating immune evasion. Spatial and pathway-specific analyses further revealed predominant activation of Trail and hypoxia pathways in NUhighepi cells, whereas NUlowepi cells were associated with estrogen signaling. A recent study elucidated how hypoxia-driven extracellular acidification impairs T cell proliferation and cytotoxicity (49). Collectively, nucleotide metabolism influences tumor microenvironments by orchestrating intercellular communication and pathway dependencies in cancer cells.

Our findings that NUhighepi cells exhibit elevated nucleotide metabolism and create an immunosuppressive microenvironment provide a strong rationale for exploring combination therapies targeting both nucleotide metabolism and immune checkpoint pathways. Studies have indicated that enzymes involved in nucleotide synthesis are highly druggable targets (6), with therapeutic agents comprising inhibitors of dihydrofolate reductase, thymidylate synthase, dihydroorotate dehydrogenase, inosine monophosphate dehydrogenase, and ribonucleotide reductase. Preclinical evidence suggests that inhibitors such as 5-fluorouracil (5-FU) and pemetrexed not only directly target rapidly proliferating cancer cells but also enhance antitumor immunity through multiple mechanisms (50): promoting immunogenic cell death and increasing tumor antigen presentation, reducing immunosuppressive adenosine accumulation, and enhancing T-cell infiltration by modulating the metabolic landscape (51). Specifically, 5-FU suppresses thymidylate synthase activity by competing with deoxyuridine monophosphate (dUMP), though dUMP accumulation may diminish its efficacy, whereas pemetrexed circumvents this limitation via competitive inhibition at the folate-binding domain independent of dUMP concentrations. While the detailed mechanisms of methotrexate, 5-FU, and pemetrexed require further clarification, their clinical benefits underscore their importance across cancer treatments. Furthermore, our investigations have identified emerging candidates like linifanib, rutin, sirolimus, PI-103, and panobinostat that target nucleotide metabolism, and the NMRS signature serves as a valuable biomarker for identifying patients with high nucleotide metabolism activity who may benefit from such combination approaches. Our findings indicate that the high-NMRS group exhibits a markedly immunosuppressive TME, distinguished by the enrichment of tumor−associated macrophages (TAMs) and Tregs, along with an elevated TIDE dysfunction score. Together, these features are consistent with an immune−excluded or desert phenotype, which is frequently linked to resistance against immune checkpoint inhibitors (ICIs). Consequently, patients classified into the high−NMRS subgroup may obtain limited benefit from ICI monotherapy. For such patients, therapeutic approaches designed to overcome immunosuppressive barriers—for example, combining ICIs with agents that target TAMs, Tregs, or angiogenesis to remodel the TME—could represent a more rational strategy and warrant further investigation. In summary, this study stratified BC into two distinct NMRS groups and elucidated their association with TME, with a specific focus on immune cell infiltration. This work provides a strategic framework for assessing prognosis and guiding therapeutic interventions in BC. Future studies could further refine the model’s utility and accuracy in evaluating immunotherapy responses by integrating additional clinical parameters and targeted drug screening.

The NMRS consists of five NMRGs: TAGLN2, PCMT1, PTMA, TUBA3D, and DCTPP1, all of which have been extensively linked to cancer. Previous studies have also demonstrated that these genes play an important role in breast cancer progression. TAGLN2 (Transgelin-2), an actin-binding protein, plays a pivotal role in cytoskeletal remodeling through its interaction with actin filaments. Elevated expression of TAGLN2 has been reported in multiple malignancies, including colorectal cancer (52), multiple myeloma (53), papillary thyroid carcinoma (54) and breast cancer (55). Functional studies establish that TAGLN2 enhances metastatic behavior in breast cancer through PI3K/AKT signaling activation (56). The protein-L-isoaspartate O-methyltransferase-1 (PCMT1), a member of the type II class of protein carboxyl methyltransferase enzymes, has been found to be involved in the repair of intracellular protein in multiple tissues via recognizing and converting L-isoaspartyl and D-aspartyl (57). Prognostic meta-analyses consistently link PCMT1 overexpression with poor survival in breast, cervical and liver cancers (58, 59). Mechanistically, PCMT1-mediated carboxyl methylation at p53 residues 29–30 impairs tumor suppressor activity, creating an environment permissive for uncontrolled growth (60). In breast cancer specifically, PCMT1 serves as both a prognostic indicator and immune microenvironment modulator (61). Our experimental data validate PCMT1 upregulation in breast cancer systems. Although PTMA and TUBA3D remain understudied in mammary tumorigenesis, our findings reveal their marked overexpression, suggesting potential biological significance requiring further investigation.

The enzyme deoxycytidine triphosphate pyrophosphatase 1 (DCTPP1) serves as a crucial hydrolase in nucleotide metabolism, facilitating the clearance of aberrant dCTP and preserving deoxyribonucleoside triphosphate (dNTP) pool equilibrium (62). DCTPP1 mediates the metabolic conversion from dCTP to dCMP in pyrimidine biosynthesis. A coordinated enzymatic network comprising CMPK1/2, NME proteins, and AK9 collaborates with DCTPP1 to maintain intracellular homeostasis of deoxycytidine phosphates (63, 64). Furthermore, targeted suppression of DCTPP1 triggers extensive reprogramming of amino acid metabolism, underscoring its pleiotropic regulatory function. In this research, we found that DCTPP1, the key gene in the signature model, was associated with poor prognosis in breast cancer patients. The knockdown of DCTPP1 inhibited the proliferation of breast cancer cells. In line with our findings, another recent study found that DCTPP1, which is activated by FOXA1, promotes the progression of breast cancer and affects cisplatin sensitivity (65). These findings underscore the significant role that DCTPP1 and nucleotide metabolism play in promoting the progression of breast cancer.

While this study reveals encouraging results, it also recognizes several constraints, most notably the limited number of samples included. The research concentrated principally on examining the biological properties of NUhighepi cells in an oncological context. Owing to the fact that only one category of tumor was analyzed, the breadth of the investigation remains considerably restricted. Consequently, the applicability of these outcomes to other forms of cancer remains questionable. Moreover, it remains uncertain whether NUhighepi constitutes an evolutionarily conserved cellular subpopulation that is common across various cancer types, a fact that adds to the existing reservations. The absence of definitive evidence at single-cell resolution regarding such epithelial cells emphasizes the continued demand for additional investigation in this domain. Furthermore, although the predictive system was developed from multiple yet limited ICI cohorts, further validation in larger immunotherapy cohorts, particularly in BC patients treated with ICIs, is still needed. Additionally, further functional experiments will be necessary to elucidate the biological role of the DCTPP1 gene in BC progression and to determine whether it can be targeted to enhance the effectiveness of immunotherapies and chemotherapies.

Looking ahead, a more comprehensive investigation into the conserved features of NUhighepi across a wide range of cancer types would be a valuable next step. This effort could involve expanding the sample size, incorporating diverse tumor types, and conducting integrative analyses that encompass both single-cell resolution and spatial dimensions. Furthermore, employing *in vivo* models represents a strategic approach to elucidate the functional contributions of NUhighepi within the wider scope of tumor biology. These investigations are anticipated to significantly advance our comprehension of its role in oncogenesis and have the potential to identify novel therapeutic targets and prognostic biomarkers applicable to diverse cancer types.

## 5 Conclusion

This study demonstrates that the prognostic signature associated with nucleotide metabolism demonstrates significant correlations with clinical outcomes, tumor progression, and immune microenvironment alterations in BC. These findings offer critical insights for formulating novel therapeutic and diagnostic strategies aimed at early intervention and prognostic assessment in BC.

## Data Availability

The original contributions presented in the study are included in the article/**Supplementary Material**. Further inquiries can be directed to the corresponding author.

## Supplementary material

Supplementary Figure 1 Spatial cell communication. **(A)** The ranking plot displayed the top 10 significant ligand-receptor pairs within the spots. **(B)** The interaction heatmap visualized the intensity of intercellular interactions mediated by the LGALS1-ITGB1, COL1A2-ITGB1and COL1A1-ITGB1 ligand-receptor pairs. **(C)** The interaction strength of the LGALS1-ITGB1, COL1A2-ITGB1and COL1A1-ITGB1 ligand-receptor pairs were represented its statistical value was shown for each spot. **(D, E)** Chord plot and network diagram depicted the spatial interactions among different cell types in the LGALS1-ITGB1, COL1A2-ITGB1and COL1A1-ITGB1 ligand-receptor pairs.

Supplementary Figure 2 Pan‐cancer analysis of NUhighepi cells. **(A, B)** UMAP plots showing cell subpopulation identification in non-small cell lung cancer (NSCLC), ovarian cancer (OV), liver hepatocellular carcinoma (LIHC), and lung squamous cell carcinoma (LSCC). **(C)** UMAP visualization of NUhighepi cells, showing enrichment in malignant cells.

## References

[1] Siegel RL Miller KD Mbbs NSW Dvm AJ . Cancer statistics. (2023) CA: a cancer journal for clinicians. 73:17–48. doi: 10.3322/caac.21763, PMID: 36633525

[2] Waks AG Winer EP . Breast cancer treatment: A review. JAMA. (2019) 321:288. doi: 10.1001/jama.2018.19323, PMID: 30667505

[3] Finley LWS . What is cancer metabolism? Cell. (2023) 186:1670–88. doi: 10.1016/j.cell.2023.01.038, PMID: 36858045 PMC10106389

[4] Hanahan D Weinberg RA . Hallmarks of cancer: the next generation. Cell. (2011) 144:646–74. doi: 10.1016/j.cell.2011.02.013, PMID: 21376230

[5] Robinson AD Eich M-L Varambally S . Dysregulation of *de novo* nucleotide biosynthetic pathway enzymes in cancer and targeting opportunities. Cancer Lett. (2020) 470:134–40. doi: 10.1016/j.canlet.2019.11.013, PMID: 31733288

[6] Mullen NJ Singh PK . Nucleotide metabolism: a pan-cancer metabolic dependency. Nat Rev Cancer. (2023) 23:275–94. doi: 10.1038/s41568-023-00557-7, PMID: 36973407 PMC10041518

[7] Peters GJ Backus HHJ Freemantle S Van Triest B Codacci-Pisanelli G van der Wilt CL . Induction of thymidylate synthase as a 5-fluorouracil resistance mechanism. Biochim Biophys Acta (BBA) - Mol Basis Dis. (2002) 1587:194–205. doi: 10.1016/S0925-4439(02)00082-0, PMID: 12084461

[8] Pavlova NN Thompson CB . The emerging hallmarks of cancer metabolism. Cell Metab. (2016) 23:27–47. doi: 10.1016/j.cmet.2015.12.006, PMID: 26771115 PMC4715268

[9] Burhans WC Weinberger M . DNA replication stress, genome instability and aging. Nucleic Acids Res. (2007) 35:7545–56. doi: 10.1093/nar/gkm1059, PMID: 18055498 PMC2190710

[10] Liu Y-C Li F Handler J Huang CRL Xiang Y Neretti N . Global regulation of nucleotide biosynthetic genes by c-myc. PloS One. (2008) 3:e2722. doi: 10.1371/journal.pone.0002722, PMID: 18628958 PMC2444028

[11] Mastelic-Gavillet B Navarro Rodrigo B Décombaz L Wang H Ercolano G Ahmed R . Adenosine mediates functional and metabolic suppression of peripheral and tumor-infiltrating CD8+ T cells. J Immunother Cancer. (2019) 7:257. doi: 10.1186/s40425-019-0719-5, PMID: 31601268 PMC6788118

[12] Nowak AK Lake RA Marzo AL Scott B Heath WR Collins EJ . Induction of tumor cell apoptosis *in vivo* increases tumor antigen cross-presentation, cross-priming rather than cross-tolerizing host tumor-specific CD8 T cells. J Immunol. (2003) 170:4905–13. doi: 10.4049/jimmunol.170.10.4905, PMID: 12734333

[13] Brown KK Spinelli JB Asara JM Toker A . Adaptive reprogramming of *de novo* pyrimidine synthesis is a metabolic vulnerability in triple-negative breast cancer. Cancer Discov. (2017) 7:391–9. doi: 10.1158/2159-8290.CD-16-0611, PMID: 28255083 PMC5380483

[14] Zhang Y Parmigiani G Johnson WE . ComBat-seq: batch effect adjustment for RNA-seq count data. NAR Genomics Bioinf. (2020) 2:lqaa078. doi: 10.1093/nargab/lqaa078, PMID: 33015620 PMC7518324

[15] Hao Y Hao S Andersen-Nissen E Mauck WM Zheng S Butler A . Integrated analysis of multimodal single-cell data. Cell. (2021) 184:3573–3587.e29. doi: 10.1016/j.cell.2021.04.048, PMID: 34062119 PMC8238499

[16] Jin S Guerrero-Juarez CF Zhang L Chang I Ramos R Kuan C-H . Inference and analysis of cell-cell communication using CellChat. Nat Commun. (2021) 12:1088. doi: 10.1038/s41467-021-21246-9, PMID: 33597522 PMC7889871

[17] Trapnell C Cacchiarelli D Grimsby J Pokharel P Li S Morse M . The dynamics and regulators of cell fate decisions are revealed by pseudotemporal ordering of single cells. Nat Biotechnol. (2014) 32:381–6. doi: 10.1038/nbt.2859, PMID: 24658644 PMC4122333

[18] Gao R Bai S Henderson YC Lin Y Schalck A Yan Y . Delineating copy number and clonal substructure in human tumors from single-cell transcriptomes. Nat Biotechnol. (2021) 39:599–608. doi: 10.1038/s41587-020-00795-2, PMID: 33462507 PMC8122019

[19] Gulati GS Sikandar SS Wesche DJ Manjunath A Bharadwaj A Berger MJ . Single-cell transcriptional diversity is a hallmark of developmental potential. Science. (2020) 367:405–11. doi: 10.1126/science.aax0249, PMID: 31974247 PMC7694873

[20] Barkley D Moncada R Pour M Liberman DA Dryg I Werba G . Cancer cell states recur across tumor types and form specific interactions with the tumor microenvironment. Nat Genet. (2022) 54:1192–201. doi: 10.1038/s41588-022-01141-9, PMID: 35931863 PMC9886402

[21] Cable DM Murray E Zou LS Goeva A Macosko EZ Chen F . Robust decomposition of cell type mixtures in spatial transcriptomics. Nat Biotechnol. (2022) 40:517–26. doi: 10.1038/s41587-021-00830-w, PMID: 33603203 PMC8606190

[22] Pham D Tan X Xu J Grice LF Lam PY Raghubar A . stLearn: integrating spatial location, tissue morphology and gene expression to find cell types, cell-cell interactions and spatial trajectories within undissociated tissues. (2020). doi: 10.1101/2020.05.31.125658

[23] Liu Z Liu L Weng S Guo C Dang Q Xu H . Machine learning-based integration develops an immune-derived lncRNA signature for improving outcomes in colorectal cancer. Nat Commun. (2022) 13:816. doi: 10.1038/s41467-022-28421-6, PMID: 35145098 PMC8831564

[24] Ma D Jiang Y-Z Liu X-Y Liu Y-R Shao Z-M . Clinical and molecular relevance of mutant-allele tumor heterogeneity in breast cancer. Breast Cancer Res Treat. (2017) 162:39–48. doi: 10.1007/s10549-017-4113-z, PMID: 28093659

[25] Mroz EA Rocco JW . MATH, a novel measure of intratumor genetic heterogeneity, is high in poor-outcome classes of head and neck squamous cell carcinoma. Oral Oncol. (2013) 49:211–5. doi: 10.1016/j.oraloncology.2012.09.007, PMID: 23079694 PMC3570658

[26] Mroz EA Tward AM Hammon RJ Ren Y Rocco JW . Intra-tumor genetic heterogeneity and mortality in head and neck cancer: analysis of data from the cancer genome atlas. PloS Med. (2015) 12:e1001786. doi: 10.1371/journal.pmed.1001786, PMID: 25668320 PMC4323109

[27] Yoshihara K Shahmoradgoli M Martínez E Vegesna R Kim H Torres-Garcia W . Inferring tumour purity and stromal and immune cell admixture from expression data. Nat Commun. (2013) 4:2612. doi: 10.1038/ncomms3612, PMID: 24113773 PMC3826632

[28] Jiang P Gu S Pan D Fu J Sahu A Hu X . Signatures of T cell dysfunction and exclusion predict cancer immunotherapy response. Nat Med. (2018) 24:1550–8. doi: 10.1038/s41591-018-0136-1, PMID: 30127393 PMC6487502

[29] Mariathasan S Turley SJ Nickles D Castiglioni A Yuen K Wang Y . TGFβ attenuates tumour response to PD-L1 blockade by contributing to exclusion of T cells. Nature. (2018) 554:544–8. doi: 10.1038/nature25501, PMID: 29443960 PMC6028240

[30] Hugo W Zaretsky JM Sun L Song C Moreno BH Hu-Lieskovan S . Genomic and transcriptomic features of response to anti-PD-1 therapy in metastatic melanoma. Cell. (2016) 165:35–44. doi: 10.1016/j.cell.2016.02.065, PMID: 26997480 PMC4808437

[31] Riaz N Havel JJ Makarov V Desrichard A Urba WJ Sims JS . Tumor and microenvironment evolution during immunotherapy with nivolumab. Cell. (2017) 171:934–949.e16. doi: 10.1016/j.cell.2017.09.028, PMID: 29033130 PMC5685550

[32] Gide TN Quek C Menzies AM Tasker AT Shang P Holst J . Distinct immune cell populations define response to anti-PD-1 monotherapy and anti-PD-1/anti-CTLA-4 combined therapy. Cancer Cell. (2019) 35:238–255.e6. doi: 10.1016/j.ccell.2019.01.003, PMID: 30753825

[33] Braun DA Hou Y Bakouny Z Ficial M Sant’ Angelo M Forman J . Interplay of somatic alterations and immune infiltration modulates response to PD-1 blockade in advanced clear cell renal cell carcinoma. Nat Med. (2020) 26:909–18. doi: 10.1038/s41591-020-0839-y, PMID: 32472114 PMC7499153

[34] Yang C Huang X Li Y Chen J Lv Y Dai S . Prognosis and personalized treatment prediction in TP53 -mutant hepatocellular carcinoma: an in silico strategy towards precision oncology. Briefings Bioinf. (2021) 22:bbaa164. doi: 10.1093/bib/bbaa164, PMID: 32789496

[35] Rao X Huang X Zhou Z Lin X . An improvement of the 2ˆ(–delta delta CT) method for quantitative real-time polymerase chain reaction data analysis. (2014) 3:71–85., PMID: 25558171 PMC4280562

[36] Vogelstein B Papadopoulos N Velculescu VE Zhou S Diaz LA Kinzler KW . Cancer genome landscapes. Science. (2013) 339:1546–58. doi: 10.1126/science.1235122, PMID: 23539594 PMC3749880

[37] Dagogo-Jack I Shaw AT . Tumour heterogeneity and resistance to cancer therapies. Nat Rev Clin Oncol. (2018) 15:81–94. doi: 10.1038/nrclinonc.2017.166, PMID: 29115304

[38] Chen DS Mellman I . Oncology meets immunology: the cancer-immunity cycle. Immunity. (2013) 39:1–10. doi: 10.1016/j.immuni.2013.07.012, PMID: 23890059

[39] Qin S Xu L Yi M Yu S Wu K Luo S . Novel immune checkpoint targets: moving beyond PD-1 and CTLA-4. Mol Cancer. (2019) 18:155. doi: 10.1186/s12943-019-1091-2, PMID: 31690319 PMC6833286

[40] Liu S Feng L Wang Z . DCTPP1: A promising target in cancer therapy and prognosis through nucleotide metabolism. Drug Discov Today. (2025) 30:104348. doi: 10.1016/j.drudis.2025.104348, PMID: 40180312

[41] Kumar V . Adenosine as an endogenous immunoregulator in cancer pathogenesis: where to go? Purinerg Signal. (2013) 9:145–65. doi: 10.1007/s11302-012-9349-9, PMID: 23271562 PMC3646124

[42] Vander Heiden MG DeBerardinis RJ . Understanding the intersections between metabolism and cancer biology. Cell. (2017) 168:657–69. doi: 10.1016/j.cell.2016.12.039, PMID: 28187287 PMC5329766

[43] Cader MZ De Almeida Rodrigues RP West JA Sewell GW Md-Ibrahim MN Reikine S . FAMIN is a multifunctional purine enzyme enabling the purine nucleotide cycle. Cell. (2020) 180:278–295.e23. doi: 10.1016/j.cell.2019.12.017, PMID: 31978345 PMC6978800

[44] Barrett RL Puré E . Cancer-associated fibroblasts and their influence on tumor immunity and immunotherapy. eLife. (2020) 9:e57243. doi: 10.7554/eLife.57243, PMID: 33370234 PMC7769568

[45] Nagl L Horvath L Pircher A Wolf D . Tumor endothelial cells (TECs) as potential immune directors of the tumor microenvironment – new findings and future perspectives. Front Cell Dev Biol. (2020) 8:766. doi: 10.3389/fcell.2020.00766, PMID: 32974337 PMC7466447

[46] Hoft SG Pherson MD DiPaolo RJ . Discovering immune-mediated mechanisms of gastric carcinogenesis through single-cell RNA sequencing. Front Immunol. (2022) 13:902017. doi: 10.3389/fimmu.2022.902017, PMID: 35757757 PMC9231461

[47] Georganaki M Van Hooren L Dimberg A . Vascular targeting to increase the efficiency of immune checkpoint blockade in cancer. Front Immunol. (2018) 9:3081. doi: 10.3389/fimmu.2018.03081, PMID: 30627131 PMC6309238

[48] Motz GT Santoro SP Wang L-P Garrabrant T Lastra RR Hagemann IS . Tumor endothelium FasL establishes a selective immune barrier promoting tolerance in tumors. Nat Med. (2014) 20:607–15. doi: 10.1038/nm.3541, PMID: 24793239 PMC4060245

[49] Jing X Yang F Shao C Wei K Xie M Shen H . Role of hypoxia in cancer therapy by regulating the tumor microenvironment. Mol Cancer. (2019) 18:157. doi: 10.1186/s12943-019-1089-9, PMID: 31711497 PMC6844052

[50] An M Mehta A Min BH Heo YJ Wright SJ Parikh M . Early immune remodeling steers clinical response to first-line chemoimmunotherapy in advanced gastric cancer. Cancer Discov. (2024) 14:766–85. doi: 10.1158/2159-8290.CD-23-0857, PMID: 38319303 PMC11061611

[51] Van Der Kraak L Goel G Ramanan K Kaltenmeier C Zhang L Normolle DP . 5-Fluorouracil upregulates cell surface B7-H1 (PD-L1) expression in gastrointestinal cancers. J Immunother Cancer. (2016) 4:65. doi: 10.1186/s40425-016-0163-8, PMID: 27777774 PMC5067917

[52] Zhang Y Ye Y Shen D Jiang K Zhang H Sun W . Identification of transgelin-2 as a biomarker of colorectal cancer by laser capture microdissection and quantitative proteome analysis. Cancer Sci. (2010) 101:523–9. doi: 10.1111/j.1349-7006.2009.01424.x, PMID: 19930159 PMC11159707

[53] Su Q Long K Adu M Jiang M Li Q Wan X . Single-cell RNA sequencing reveals immune regulatory mechanisms and molecular therapeutic strategies in the microenvironment of multiple myeloma. Int J Surg. (2025). doi: 10.1097/JS9.0000000000003306, PMID: 40899849 PMC12825540

[54] Zeng C Wang F Huang Y Zhang H . CXCR7-TAGLN2 protein complex regulates invasion and metastasis in papillary thyroid carcinoma: a potential therapeutic target. Front Immunol. (2025) 16:1627419. doi: 10.3389/fimmu.2025.1627419, PMID: 41169389 PMC12568504

[55] Huang W Wang W Dong T . Telomere maintenance-related genes are essential for prognosis in breast cancer. BCTT. (2025) 17:225–39. doi: 10.2147/BCTT.S506783, PMID: 40028272 PMC11869761

[56] Liu L Meng T Zheng X Liu Y Hao R Yan Y . Transgelin 2 promotes paclitaxel resistance, migration, and invasion of breast cancer by directly interacting with PTEN and activating PI3K/akt/GSK-3β Pathway. Mol Cancer Ther. (2019) 18:2457–68. doi: 10.1158/1535-7163.MCT-19-0261, PMID: 31488699

[57] DeVry CG Clarke S . Polymorphic forms of the protein L-isoaspartate (D-aspartate) O-methyltrans-ferase involved in the repair of age-damaged proteins. J Hum Genet. (1999) 44:275–88. doi: 10.1007/s100380050161, PMID: 10496068

[58] Shan L Wang X Li Y Li L Wu S Xi X . Elevated expression of protein-L-isoaspartate O-methyltransferase-1 (PCMT1) in cervical cancer. Transl Cancer Res TCR. (2022) 11:2582–90. doi: 10.21037/tcr-21-2700, PMID: 36093534 PMC9459589

[59] Liu J Liu B Li Y Mi Z Tan H Rong P . PCMT1 is a potential target related to tumor progression and immune infiltration in liver cancer. Eur J Med Res. (2023) 28:289. doi: 10.1186/s40001-023-01216-1, PMID: 37596654 PMC10436427

[60] Lee J-C Kang S-U Jeon Y Park JW You J-S Ha S-W . Protein L-isoaspartyl methyltransferase regulates p53 activity. Nat Commun. (2012) 3:927. doi: 10.1038/ncomms1933, PMID: 22735455 PMC3621463

[61] Guo J Du X Li C . PCMT1 is a potential prognostic biomarker and is correlated with immune infiltrates in breast cancer. BioMed Res Int. (2022) 2022:4434887. doi: 10.1155/2022/4434887, PMID: 35535040 PMC9078795

[62] Ráez-Meseguer C Amengual-Tugores AM Forteza-Genestra MA Orvay-Pintos F Gomila RM Martorell-Crespí G . Comparative analysis of platelet-derived extracellular vesicle protein extraction methodologies for mass spectrometry. J Proteome Res. (2025) 24:3931–42. doi: 10.1021/acs.jproteome.5c00089, PMID: 40691760 PMC12323001

[63] Rampazzo C Tozzi MG Dumontet C Jordheim LP . The druggability of intracellular nucleotide-degrading enzymes. Cancer Chemother Pharmacol. (2016) 77:883–93. doi: 10.1007/s00280-015-2921-6, PMID: 26614508

[64] Proust B Herak Bosnar M Ćetković H Tokarska-Schlattner M Schlattner U . Mitochondrial NME6: A paradigm change within the NME/NDP kinase protein family? Cells. (2024) 13:1278. doi: 10.3390/cells13151278, PMID: 39120309 PMC11312278

[65] He Y Li G Shi X Bie J Du G Feng X . DCTPP1 is transcriptionally activated by FOXA1 to affect cisplatin sensitivity in triple-negative breast cancer via suppression of ferroptosis. Cell Biochem Biophys. (2025) 83:4755–68. doi: 10.1007/s12013-025-01801-7, PMID: 40549289

