# Supplementary figures and images for: Spatial transcriptome and single-cell sequencing reveal the role of nucleotide metabolism in breast cancer progression and tumor microenvironment
Source: Front Oncol. 2026 Jan 14;15:1703778. doi: 10.3389/fonc.2025.1703778 (PMC12847018; doi:10.3389/fonc.2025.1703778)

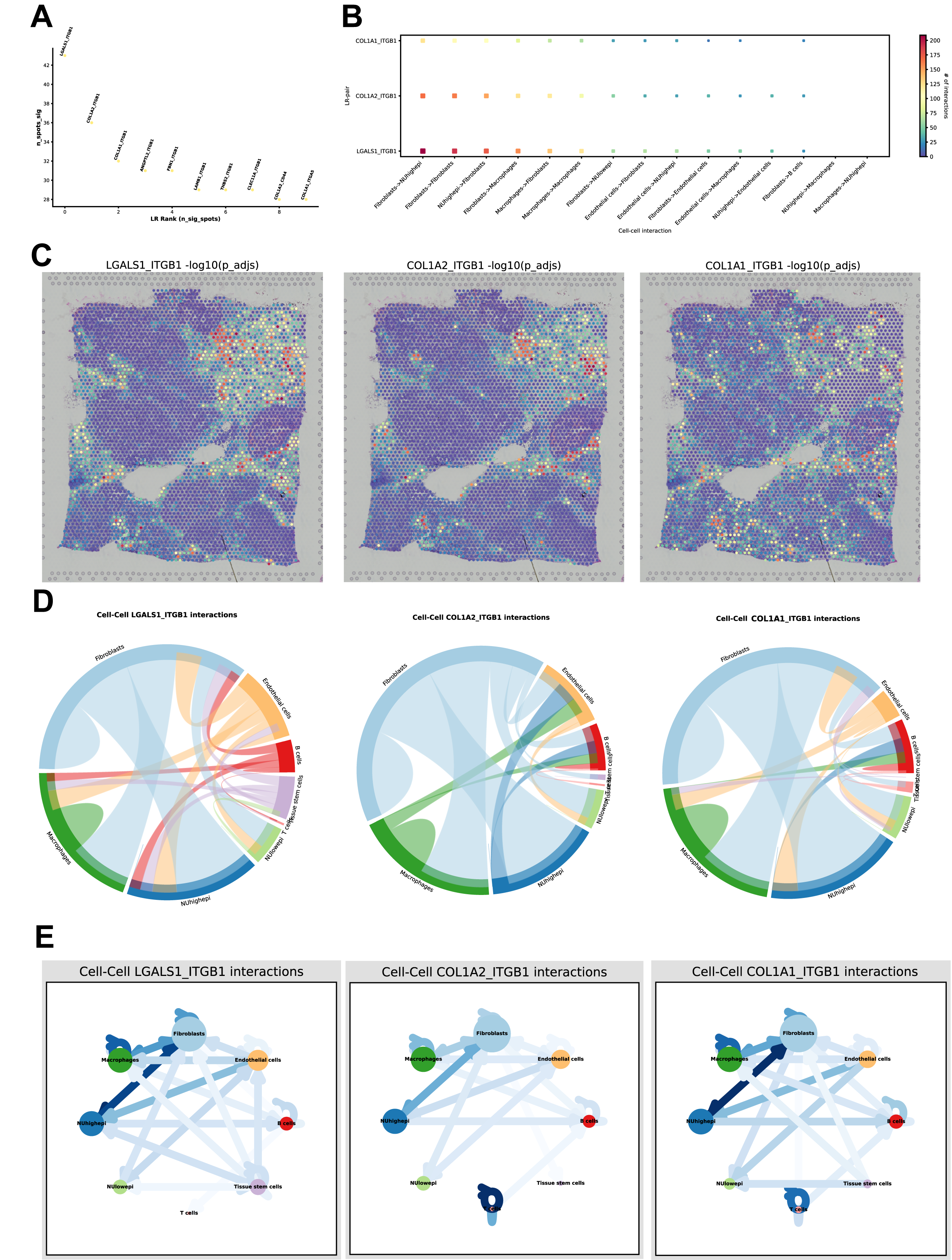

Supplement: Supplementary Figure 1 — Spatial cell communication. (A) The ranking plot displayed the top 10 significant ligand-receptor pairs within the spots. (B) The interaction heatmap visualized the intensity of intercellular interactions mediated by the LGALS1-ITGB1, COL1A2-ITGB1and COL1A1-ITGB1 ligand-receptor pairs. (C) The interaction strength of the LGALS1-ITGB1, COL1A2-ITGB1and COL1A1-ITGB1 ligand-receptor pairs were represented its statistical value was shown for each spot. (D, E) Chord plot and network diagram depicted the spatial interactions among different cell types in the LGALS1-ITGB1, COL1A2-ITGB1and COL1A1-ITGB1 ligand-receptor pairs. [file Image1.tiff]

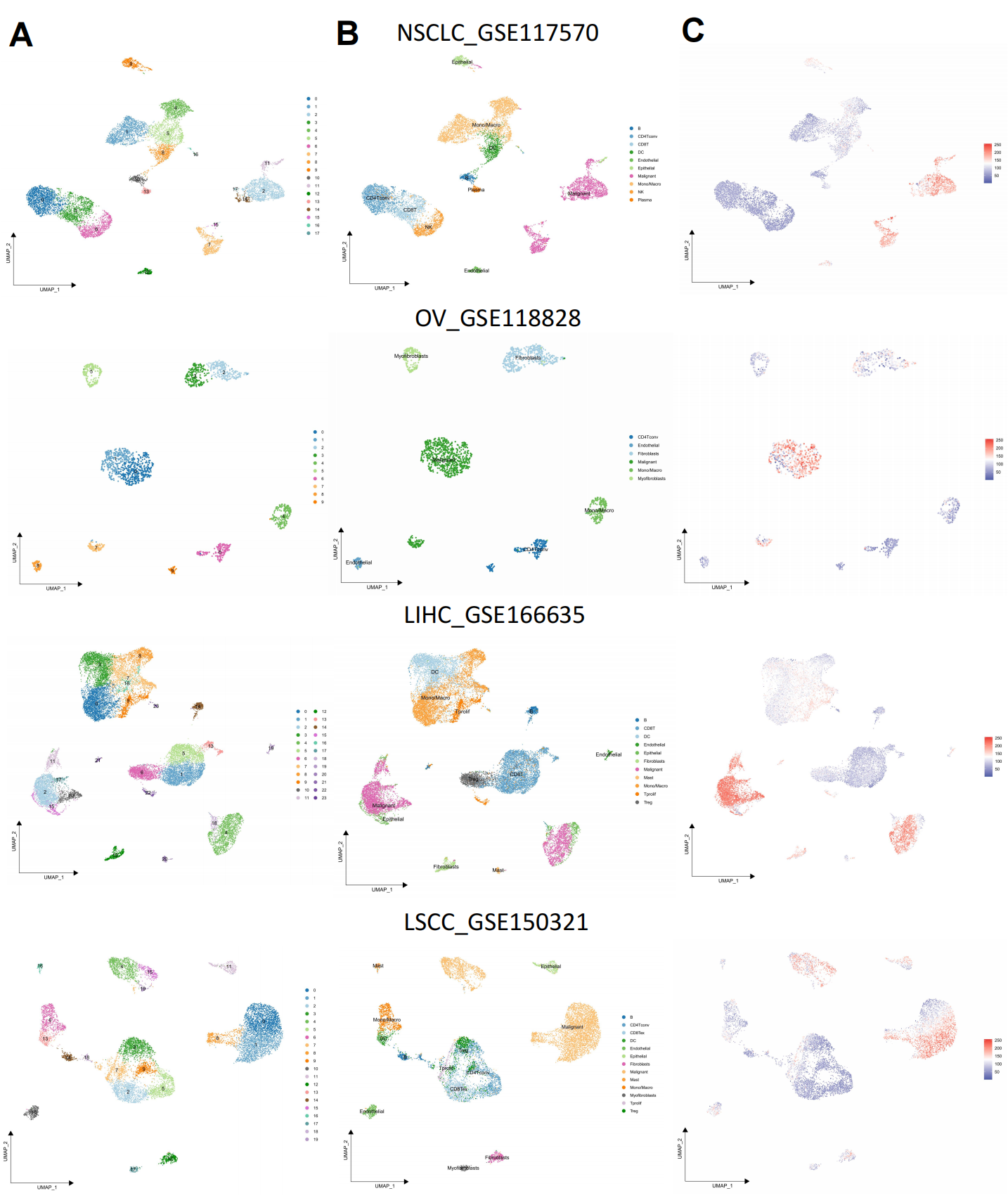

Supplement: Supplementary Figure 2 — Pan‐cancer analysis of NUhighepi cells. (A, B) UMAP plots showing cell subpopulation identification in non-small cell lung cancer (NSCLC), ovarian cancer (OV), liver hepatocellular carcinoma (LIHC), and lung squamous cell carcinoma (LSCC). (C) UMAP visualization of NUhighepi cells, showing enrichment in malignant cells. [file Image2.tif]
